# Quorum‐dependent transfer of the opine‐catabolic plasmid pAoF64/95 is regulated by a novel mechanism involving inhibition of the TraR antiactivator TraM

**DOI:** 10.1002/mbo3.625

**Published:** 2018-04-10

**Authors:** Margaret E. Wetzel, Robert E. Asenstorfer, Max E. Tate, Stephen K. Farrand

**Affiliations:** ^1^ Department of Microbiology The University of Illinois at Urbana‐Champaign Urbana IL USA; ^2^ School of Agriculture, Food and Wine The University of Adelaide Osmond SA Australia

**Keywords:** agrobacterium, bacterial plasmids, gene regulation, quorum‐sensing

## Abstract

We previously described a plasmid of *Agrobacterium spp*., pAoF64/95, in which the quorum‐sensing system that controls conjugative transfer is induced by the opine mannopine. We also showed that the quorum‐sensing regulators TraR, TraM, and TraI function similarly to their counterparts in other *repABC* plasmids. However, *traR*, unlike its counterpart on Ti plasmids, is monocistronic and not located in an operon that is inducible by the conjugative opine. Here, we report that both *traR* and *traM* are expressed constitutively and not regulated by growth with mannopine. We report two additional regulatory genes, *mrtR* and *tmsP*, that are involved in a novel mechanism of control of TraR activity. Both genes are located in the distantly linked region of pAoF64/95 encoding mannopine utilization. MrtR, in the absence of mannopine, represses the four‐gene *mocC* operon as well as *tmsP*, which is the distal gene of the eight‐gene *motA* operon. As judged by a bacterial two‐hybrid analysis, TmsP, which shows amino acid sequence relatedness with the TraM‐binding domain of TraR, interacts with the antiactivator. We propose a model in which mannopine, acting through the repressor MrtR, induces expression of TmsP which then titrates the levels of TraM thereby freeing TraR to activate the *tra* regulon.

## 1 INTRODUCTION

Many members of the family Rhizobiales harbor very large extrachromosomal elements. Some of these plasmids, which replicate using a conserved *repABC* system, confer on their host bacteria defining biological functions. For example, induction of crown gall tumors and hairy roots on susceptible plants by the agrobacterial group is conferred by Ti (tumor‐inducing) and Ri (hairy root‐inducing) plasmids (reviewed in Tempé, Petit, & Farrand, 1984), while the capacity to nodulate and fix nitrogen on legumes by members of the rhizobial group is conferred by Sym (symbiotic) plasmids (Cevallos, 2002; Galibert et al., 2001). Characteristically, these plasmids contain a conserved core of replication functions that can stably replicate large amounts of cargo DNA at a low copy number (Cevallos, Cervantes‐Rivera, & Gutiérrez‐Ríos, 2008; Pinto, Pappas, & Winans, 2012). All of the known Ti, Ri, and Ao (opine‐catabolic) plasmids, as well as a few megaplasmids of unknown function characterized to date encode Class I conjugative transfer systems composed of an IncP‐like type 4 secretion system (T4SS), an IncQ‐like *oriT*, and an enhanced IncQ‐like *trans*‐acting DNA metabolism system (Cook & Farrand, 1992; Ding & Hynes, 2009; Farrand, Hwang, & Cook, 1996; Li, Everhart, & Farrand, 1998). The components of the T4SS are encoded by the *traI‐trb* operon, which is invariably adjacent and divergently oriented to the *repABC* operon responsible for replication and partitioning of the plasmid (Figure S1; Li et al., 1998; Wetzel, Olsen, Chakravartty, & Farrand, 2015)]. The DNA metabolism functions are encoded by two operons, *traAFBH* and *traCDG* that are divergently oriented and separated by a ca 250 bp region that encodes the *cis*‐acting *oriT* site (Figure S1; Cook & Farrand, 1992; Farrand et al., 1996; Wetzel et al., 2015). Consistent with this genetic suite, a number of these plasmids are known to be self‐conjugative; of the agrobacterial plasmids, the most well‐studied are two Ti plasmids, pTiC58 and pTiR10, and two Ao plasmids, pAtK84b and pAoF64/95 (Ellis, Kerr, Petit, & Tempé, 1982; Fuqua & Winans, 1994; Oger & Farrand, 2002; Petit & Tempé, 1978; Wetzel, Kim, Miller, Olsen, & Farrand, 2014).

In these four cases, conjugation is strongly controlled, with expression of the transfer genes requiring two exogenous signals (Fuqua & Winans, 1996b; Oger & Farrand, 2002; Piper, Beck von Bodman, Hwang, & Farrand, 1999; Wetzel et al., 2014). The first is a particular opine, a type of novel carbon compound produced by the crown gall tumors induced by pathogenic members of the two genera. Opines serve as novel and specific carbon sources to the bacteria that induce the neoplasias because genes encoding the catabolic functions are invariably located on the Ti plasmid. For example, strains of *A. tumefaciens* harboring pTiC58 induce tumors that produce two opine types, nopaline, an imine conjugate of arginine and alpha‐keto glutarate, and agrocinopines A and B, phosphodiesters of sucrose or fructose and arabinose (reviewed in Dessaux, Petit, Farrand, & Murphy, 1998; Farrand, 1998). Growth with the agrocinopines, but not nopaline induces transfer of this Ti plasmid to bacterial recipients (Ellis et al., 1982). Similarly, strains harboring pTiR10 induce tumors that produce octopine, a conjugate of arginine and pyruvate, and the four mannityl opines, conjugates of mannose and glutamate or glutamine, and transfer is induced by growth with the former but not the latter family (Fuqua & Winans 1994 and reviewed in Dessaux et al. 1998 and Farrand 1998).

The second signal is an acyl‐homoserine lactone (acyl‐HSL) produced by the bacteria themselves (Zhang, Murphy, Kerr, & Tate, 1993). This quorum‐sensing (QS) signal is synthesized by TraI, the product of the first gene of the *traI‐trb* operon (Figure S1; Hwang et al., 1994; Li et al., 1998). This signal is recognized and bound by the LuxR‐type QS activator TraR which then dimerizes and binds to 18 bp inverted repeats, called *tra* boxes, located in the promoter regions of the *trb* and *tra* operons (Figure S1; Fuqua & Winans, 1994; Fuqua & Winans, 1996a; Luo & Farrand, 1999; Piper, Beck von Bodman, & Farrand, 1993; Zhu & Winans, 1999). *traR* is invariably located close to the two *tra* operons (Wetzel et al., 2015). An additional component of the regulatory system, TraM, functions as an antiactivator by directly interacting with TraR thereby preventing the activator from binding to its *tra* box target sites (Figure S1; Hwang, Smyth, Luo, & Farrand, 1999; Luo, Qin, & Farrand, 2000). TraM serves to prevent inappropriate activation of the *tra* regulon by basal levels of TraR.

In the two most well‐studied plasmids, pTiC58 and pTiR10, the *traR* gene is located in an operon the expression of which is controlled by a specific opine‐responsive transcription factor (Figure S1; Fuqua & Winans, 1994; Piper et al., 1999). For example, in pTiR10, *traR* is the distal gene of the *occ* operon that encodes the proteins responsible for transport and catabolism of octopine (Cho, Fuqua, Martin, & Winans, 1996; Fuqua & Winans, 1994). This operon is controlled by OccR, a LysR‐like activator that, in turn, must bind octopine for activity (Fuqua & Winans, 1994; Habeeb, Wang, & Winans, 1991). Similarly, *traR* of pTiC58 is located in the *arc* operon adjacent and divergently oriented to the *acc* operon responsible for the transport and catabolism of agrocinopines A and B (Kim & Farrand, 1997; Piper et al., 1999). Both operons are controlled by AccR, a FucR‐like repressor (Beck von Bodman, Hayman, & Farrand, 1992; Piper et al., 1999). This regulator loses affinity for its operator sequences in the *acc* and *arc* promoter regions when it binds arabinose‐2‐phosphate, the first intermediate in catabolism of the two agrocinopine opines (El Sahili et al., 2015). In both Ti plasmids, the two signals thus form a regulatory cascade with the conjugative opine controlling expression of the quorum‐sensing system which, in turn, is responsible for inducing transcription of the *tra* regulon (Figure S1).

Although transfer of pTiR10 and pTi15955 is induced only by octopine (Fuqua & Winans, 1994), the two virtually identical plasmids contain a second allele of *traR* (Oger, Kim, Sackett, Piper, & Farrand, 1998; Zhu & Winans, 1998). This gene, called *trlR*, is a frameshift mutant and produces a truncated form of TraR that retains the N‐terminal acyl‐HSL‐binding and dimerization domains but lacks the C‐terminal DNA‐binding domain (Oger et al., 1998; Zhu & Winans, 1998). Of significance to our work, *trlR* is the distal gene of the *mot* operon that encodes an ABC‐type transporter responsible for the uptake of mannopine (MOP), one of the four mannityl opines (Kim & Farrand, 1996; Oger et al., 1998; Zhu & Winans, 1998). Expression of this operon, including *trlR*, is induced by growth of the bacteria with MOP (Oger et al., 1998; Zhu & Winans, 1998). TrlR exerts a dominant‐negative effect on TraR; co‐expression of *trlR* and *traR* mediated by growth of the donors with both octopine and mannopine results in reduced levels of transfer as compared to cells grown only with octopine (Oger et al., 1998; Zhu & Winans, 1998). This inhibitory effect results from the ability of TrlR to form inactive heterodimers with TraR (Chai, Zhu, & Winans, 2001).

Given the existence of *trlR* and its spatial and regulatory association with the *mot* operon, we reasoned that there should exist in nature Ti, Ri, or Ao plasmids in which mannopine serves as the conjugative opine. We also postulated that the functional *traR* gene of such plasmids would be associated with the *mot* operon. We previously reported on a plasmid, pAoF64/95, in which growth with MOP strongly induced conjugative transfer (Wetzel et al., 2014). This plasmid, while not encoding a T‐region or any *vir* genes, does carry the canonical Class I *repABC*,* traI‐trb* and *tra* operons as well as the *traR*‐*traM* dyad. It also carries a full set of genes for uptake and catabolism of the mannityl opines. However, *traR* is not associated with the *mot* operon and, while located in the canonical site between *traM* and the *traAFBH* operon, *traR* is, in fact, monocistronic (Wetzel et al., 2014). Mutational analysis showed that *traR* of this plasmid is essential for MOP‐dependent induction of the synthesis of the acyl‐HSL QS signal and the resultant conjugative transfer of the element (Wetzel et al., 2014). Moreover, a *traM* null mutant is constitutive for transfer indicating that the antiactivator is functional (Wetzel et al., 2014). However, while the *traR* mutation could be complemented *in trans*, growth with MOP still was required to induce transfer (Wetzel et al., 2014). These observations, coupled with the gene arrangement, led us to ask how growth with MOP induces the QS system that regulates plasmid transfer.

In this study, we show that TraR of pAoF64/95 directly activates transcription of the three promoters of the *tra* regulon in an acyl‐HSL‐dependent manner and that TraM inhibits the activity of TraR. We also demonstrate that transcription of both *traR* and *traM* is constitutive and does not respond to growth with the conjugative opine. We determine that a gene located in the *moc* regulon, we named *mrtR*, encodes a negative regulator that represses several components of this gene set including an operon that terminates in a small gene encoding a protein related to the region of TraR that interacts with TraM. The *mrtR* mutant also is constitutive for conjugative transfer. Repression of the *moc* regulon and conjugative transfer by MrtR is relieved by growth of the cells with MOP. Finally, we provide data that support a model in which the small TraR‐like protein, which we name TmsP, interacts with TraM thereby freeing up sufficient amounts of functional TraR to activate transcription of the *tra* regulon.

## 2 EXPERIMENTAL PROCEDURES

### 2.1 Strains, media, and growth conditions

Bacterial strains and plasmids used in this study are listed in Table S1. For growth in liquid, cultures of *A. tumefaciens* strain NTL4 (Luo, Clemente, & Farrand, 2001) harboring various plasmids were inoculated into either MG/L (Cangelosi, Best, Martinetti, & Nester, 1991) or AB minimal medium (Cangelosi et al., 1991) supplemented to 0.005% yeast extract and to 0.2% mannitol or to 500 μg/ml mannopine as the sole source of carbon. Mannopine was a gift from Yves Dessaux (Institut des Sciences du Végétal, Gif‐sur‐Yvette, France), while deoxy‐fructosyl glutamine (DFG) was the kind gift of Kun‐Su Kim (Sogang University, Seoul, South Korea). Samples of DFG also were synthesized essentially as described by Chilton et al. (1995). Strains of *Agrobacterium* spp. were grown on solid medium using 2% agar and either Nutrient Broth (Difco, Detroit, MI) or AB minimal (Cangelosi et al., 1991) with 0.2% mannitol (ABM) as the sole carbon source. *Escherichia coli* strains were grown using liquid or solid L broth (Fischer Scientific). Occasionally, *E. coli* was grown on MacConkey agar (Difco). Antibiotics were added to the media at the following concentrations in μg/ml: gentamicin, 25; spectinomycin, 100; streptomycin, 100; rifampicin, 50; kanamycin, 25 or 50; tetracycline, 5 or 10; carbenicillin, 100; ampicillin, 100; chloramphenicol, 34. Isopropyl‐β‐d‐thiogalactopyranoside (IPTG) was used at concentrations ranging from 0.1 to 1 mmol/L. X‐gal (5‐bromo‐4‐chloro‐3‐indolyl‐β‐d‐galactopyranoside) was used at a concentration of 40 μg/ml, while X‐gluc (5‐bromo‐4‐chloro‐3‐indolyl‐β‐d‐glucoronic acid) was used at a concentration of 50 μg/ml. When required synthetic N‐(3‐oxo‐octanoyl)‐l‐homoserine lactone (3‐oxo‐C8‐HSL, AAI, Sigma‐Aldrich, St. Louis, MO) was added at concentrations of 10 nmol/L in liquid media and 50 nmol/L in solid media unless otherwise stated. Strains of *Agrobacterium* were grown at either 28 or 30°C, while *E. coli* was grown at 37°C, or when required, 30°C.

### 2.2 Conjugative transfer efficiency

Conjugative transfer of plasmids from derivatives of NTL4 harboring transferrable plasmids into recipient strain *A. tumefaciens* C58C1RS (Table S1) was conducted by the drop plate method (Beck von Bodman, McCutchan, & Farrand, 1989; Farrand, Qin, & Oger, 2002; Piper & Farrand, 2000). Transconjugates were selected using appropriate resistance to kanamycin and/or tetracycline and donors were counterselected using rifampicin and streptomycin. Transfer frequencies are expressed as transconjugates recovered per input donor.

### 2.3 β‐galactosidase and β‐glucoronidase assays

Activity of β‐galactosidase was measured using ONPG (O‐nitrophenyl‐β‐d‐galactopyranoside) and a modified version of the assay described by Miller (Miller, 1972) or a microtiter version of the Miller assay further modified by Slauch and Silhavy (Slauch & Silhavy, 1991). β‐glucuronidase activity was assessed using PNPG (4‐nitrophenyl‐β‐d‐glucuronide) and a modified version of the assay described by Jefferson, Burgess, and Hirsh (Jefferson, Burgess, & Hirsh, 1986).

Non‐quantitative β‐galactosidase assays where carbon sources were assessed as potential inducers were performed on plates of solid AB medium supplemented with mannitol to 0.05% along with X‐gal, appropriate antibiotics, and where indicated, IPTG to 1 mmol/L. In the minimal plasmid system when pMWS109::*traAlacZ*, which does not encode *traI*, was used and AAI was not added to Whatman discs, the acyl‐homoserine lactone was added directly to the culture medium. Colonies of the strains to be tested were suspended in 250 μl volumes of 0.9% NaCl and suspensions of the cells were streaked across the plate. Sterile discs (6 mm diameter) of Whatman 3MM paper impregnated with 5 μl volumes of either 10% mannitol, 25 μg/ml mannopine, or ~25 μg/ml DFG were placed on top of the streak of the strain to be tested, the plates were incubated at 28°C, and monitored by eye for 48 hours. When no additional carbon sources were supplied on Whatman discs a similar protocol was utilized only using solid ABM supplemented with appropriate antiobiotics, either X‐gal or X‐gluc, and IPTG to 1 mmol/L, unless otherwise specified. Where designated, 2 μl volumes of 25 μmol/L AAI, or ethyl acetate were supplied on the Whatman discs.

### 2.4 Construction of *lacZ* and *uidA* gene fusions

To create a minimal plasmid system that reported *traR*‐dependent activation, we utilized TnHoHo1 (Stachel, An, Flores, & Nester, 1985) to construct a *traA*::*lacZ* fusion on the cosmid pMWS109 (Table S1). This cosmid contains both the *traCDG* and *traAFBH* operons as well as the 3′ end of the *trb* operon, along with *traR* and *traM*. Briefly, pMWS109 was transformed into *E. coli* strain S17‐1(pHoHo1, pSShe). The resulting strain was filter‐mated with recipient strain NTL4(pAoF64/95) as described previously (Farrand et al., 2002). Transconjugates were selected on ABM, a minimal medium that excludes growth of the *E. coli* donor, supplemented with tetracycline and carbenicillin. Transconjugates were screened for β‐galactosidase activity on solid AB minimal medium containing X‐gal, tetracycline, and carbenicillin, and either mannitol or mannopine as the sole source of carbon. Colonies that were white on the mannitol medium and blue on the mannopine medium were further purified and the locations of the Tn3‐*lacZ* gene fusions on pMWS109 were determined by DNA sequence analysis. One plasmid, pMWS109*traA*::*lacZ*, was identified in which *lacZ* is transcriptionally fused to *traA*.

The *lacZ* fusions to *traR*,* mrtR*, and *tmsP* that are integrated into pAoF64/95 were constructed by single cross‐overs using the methods and vectors of Kalogeraki and Winans (Kalogeraki & Winans, 1997). For each gene, two fusions were made separately, one using pVik112, which created a transcriptional fusion and left a copy of the target gene intact, and the second using pVik107, which created a translational gene fusion to *lacZ* and also disrupted the target gene. Portions of each target gene were PCR‐amplified from genomic DNA isolated from NTL4(pAoF64/95) using the primer pairs listed in Table S2. We cloned the resulting fragment into either pVik107 or pVik112 using the restriction sites labeled in the primer names and underlined in the primer sequences (Table S2). Fusions in the clones were confirmed by sequence analysis, the plasmids were transformed into appropriate *Agrobacterium* strains and integrated into pAoF64/95 as described previously (Wetzel et al., 2014). The *lacZ* translational fusion to *traM*, which also created a *traM* mutant, was constructed in a similar manner and is described in Wetzel et al. (2014).

Plasmid pL6480p*traR*::*lacZ* was constructed by cloning the 5′ intergenic region and a 5′ portion of *traR* into pLKC480 (Tiedeman & Smith, 1988) and subcloning the *traR*::*lacZY* fusion into pLAFR6, a vector that contains two terminator regions flanking the multiple cloning site (Huynh, Dahlbeck, & Staskawicz, 1989; Rahme, Mindrinos, & Panopoulos, 1991). First, the upstream promoter region and first few codons of *traR* were amplified by PCR from genomic DNA prepared from NTL4(pAoF64/95) using the primers listed in Table S2. This PCR product was then directionally ligated into the SalI and HindIII sites of pLKC480, and a correct clone was confirmed using sequence analysis. pLKC480::*traR* was then digested with BamHI and PstI and the *traR*::*lacZY* fragment was subcloned using the same restriction sites into pLAFR6 to create pL6480p*traR*::*lacZ*.

The *traC*‐*traA* intergenic region of pAoF64/95 was directionally cloned into pRG970b (Table S1) such that *ptraC* was fused to *lacZ*, and *ptraA* was fused to *uidA*. The *mocC* promoter from pAoF64/95 was fused to the *uidA* gene located on pRG970b, and the *traI*‐*repA* intergenic region of pAoF64/95 was cloned into pRG970b creating *traI*::*lacZ* and *repA*::*uidA* fusions. Each of these reporter clones was constructed by amplifying the appropriate intergenic region of pAoF64/95 using the primer pairs listed in Table S2. The PCR products, which contained BamHI and XmaI sequences were directionally ligated into pRG970b digested with the same enzymes. All fusions were verified by sequence analysis.

### 2.5 Construction of vectors expressing one or more genes

We utilized pSRKGm (Table S1) as a cloning vector to express *mrtR* and the three different versions of *tmsP* in an ITPG‐dependent manner. Each gene was amplified by PCR using total genomic DNA prepared from strain NTL4(pAoF64/95) and the amplicons were cloned into pSRKGm using the primers pairs and restriction enzyme sites labeled in the primer names and primer sequences listed in Table S2. We confirmed candidate clones by sequence analysis. Construction of pSRKGm::*traR* and pSRKGm::*traM* have been previously described (Wetzel et al., 2014).

For the bacterial two‐hybrid system, in‐frame versions of *traM* and *tmsPs* were cloned into the *lexA* fusion vectors, pSR658 and pSR659 (Daines, Granger‐Schnarr, Dimitrova, & Silver, 2002). Briefly, *traM* and *tmsPs* were separately amplified by PCR from total genomic DNA isolated from strain NTL4(pAoF64/95) using the primer pairs listed in Table S2. The PCR products, which contain the restriction sites indicated in the primer names and underlined in the primer sequences, were then directionally ligated into the appropriate vector digested with the same restriction enzymes. Clones with the correct fusions identified by sequence analysis were then transformed into *E. coli* strain Su202 (Table S1) such that Su202 contained one *lexA*::*traM* and one *lexA*::*tmsPs* fusion. We created two sets of tester strains. One contained *traM* fused to *lexA*‐WT on pSR658 and *tmsPs* fused to *lexA*‐MUT on pSR659, and the other contained *traM* fused to *lexA*‐MUT on pSR659 and *tmsPs* fused to *lexA*‐WT on pSR658.

The mannopine transport region containing the *motABCD* operon of strain 15955 (Hong, Dessaux, Chilton, & Farrand, 1993; Hong & Farrand, 1994) was amplified from total genomic DNA using the primer pairs listed in Table S2. The product was then digested with XmaI and ligated into the XmaI site of pSa152 (Table S1). Candidate clones were confirmed by sequence analysis and pSa152::*mot15955* was transformed into NTL4(pMWS109*traA*::*lacZ*, pSRKGm::*mrtR*).

### 2.6 Construction of mutant strains

Antibiotic resistance genes or cassettes were amplified by PCR using Taq DNA polymerase (New England Biolabs). Allele replacement mutations were constructed by the methods of Datsenko and Wanner (Datsenko & Wanner, 2000) using primers that contain the 5′ overhang sequences of the gene of interest shown in Table S2 and by amplifying either the kanamycin‐resistant cassette from pKD4 (Datsenko & Wanner, 2000) or the *tetA* gene from *A. tumefaciens* strain C58. Kanamycin‐marked deletion replacements of *tmsP*,* mocC*, and *mocD* were constructed by transforming the appropriate PCR product into *E. coli*(pKD46, pMWS112). Kan^R^ replacement mutations of *tmsP*,* mocC*, or *mocD* in pMWS112 were selected using resistance to kanamycin. The mutations were confirmed by restriction digests and PCR fragment analysis of the regions upstream and downstream of the three genes using primers shown in Table S2. Derivatives of cosmid pMWS112 (Table S1) containing correct mutations in *mocC* or *mocD* were subsequently digested with XhoI and the fragments bearing the mutant alleles were separately subcloned into pWM91 (Table S1). The resulting plasmids were used to individually recombine the mutant alleles into pAoF64/95 harbored by NTL4 as described previously (Wetzel et al., 2014). The kanamycin‐marked deletion derivative of *tmsP* was similarly recombined into pAoF64/95 by subcloning the appropriate fragment into pWM91, only using BamHI and XhoI to digest pMWS112Δ*tmsP* and pWM91.

Considering that the *tmsP*::*lacZ* fusions and the *tmsP* mutant all code for resistance to kanamycin, constructing a *mrtR* deletion derivative of these strains involved a different antibiotic and therefore a different strategy. First, we cloned an ApaI fragment of pMSW112 that included *mrtR* into pAW19 (Table S1), a derivative of pWM91 that codes for resistant to kanamycin. The resulting construct, pAW19::112, was subsequently transformed into *E. coli* (pJW106) (Table S1) (Quick, Shah, & Wilson, 2010). We then PCR‐amplified the native *tetA* gene from total genomic DNA of strain C58 using primers that contained 5′ overhangs of *mrtR* (Table S2). The resulting PCR product was transformed into *E. coli*(pJW106, pAW19::112) and the *mrtR*::*tetA* indel mutation was selected using resistance to tetracycline. The mutation was confirmed, and the tetracycline‐marked mutant of *mrtR* was moved into strains NTL4(pAoF64/95Δ*tmsP*), NTL4(pAoF64/95Δ*tmsP*::*lacZ*), and NTL4(pAoF64/95*tmsP*::*lacZ*) using the methods previously described by selecting for resistance to both kanamycin, for the original mutation or gene fusion, and tetracycline (Wetzel et al., 2014). The construction of the *traM*,* traR*, and *mrtR* mutants has been described previously (Wetzel et al., 2014; Wetzel et al., 2015).

## 3 RESULTS

### 3.1 TraR activates the *traA, traC, and traI* promoters in an AAI‐dependent manner

**Figure 1:**
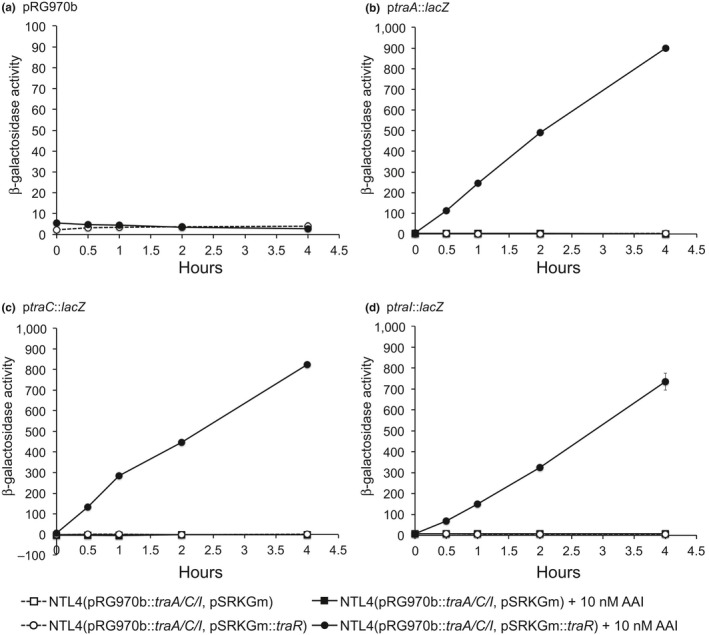
*traR* activates the *traA*,* traC*, and *traI* promoters in an AAI‐dependent manner. Cells of each bacterial strain were grown in ABM medium to an approximate OD_600_ of 0.4 and the cultures were divided into two equal volumes. AAI dissolved in ethyl acetate was added to 10 nmol/L to one portion, while the second portion received the same volume of ethyl acetate and the cultures were re‐incubated. Samples were collected immediately and after periods of 0.5, 1, 2, and 4 hours. All samples were assessed for β‐galactosidase activity as described in Experimental Procedures. (a) Strain NTL4(pRG970b, pSRKGm::*traR*). Strain NTL4(pSRKGm::*traR*) or NTL4(pSRKGm) harboring pRG970b with transcriptional fusions of *lacZ* to the promoters of (b) *traA*, (c) *traC*, and (d) *traI*. All media contained 1 mmol/L IPTG to induce expression of *traR*. Each experiment was done two times with three internal repeats. Shown for each figure n = 1 and the error bars are the standard deviations of the three internal repeats

We previously reported that a mutation in *traR* of pAoF64/95 abolished the ability of a donor strain to transfer the plasmid (Wetzel et al., 2014). To assess if TraR directly activates the *traI*/*trb* operon and the two *tra* operons, we individually cloned the *traA*,* traC,* and *traI* promoters into pRG970b, creating *lacZ* transcriptional fusions to each promoter. The resulting three plasmids were transformed into the Ti‐plasmidless strain NTL4 (Table S1). We subsequently added either empty vector pSRKGm or pSRKGm containing a cloned copy of *traR in trans* to this reporter system. The cells were grown to exponential phase in minimal media with IPTG to induce expression of *traR*, each of the six cultures were then split into two, and AAI was added to one flask of each of the six cultures. Samples were taken periodically and β‐galactosidase activity was assessed. The *traA*,* traC,* and *traI* promoters were strongly induced only when TraR was expressed and AAI was added to the culture medium (Figure 1b–d, black circles). β‐galactosidase activity was very low in cells lacking TraR (white and black squares in Figure 1b–d) and when AAI was not added to the culture medium (Figure 1 white circles and white squares).

### 3.2 TraM inhibits TraR‐mediated activation of a *tra* promoter

**Figure 2:**
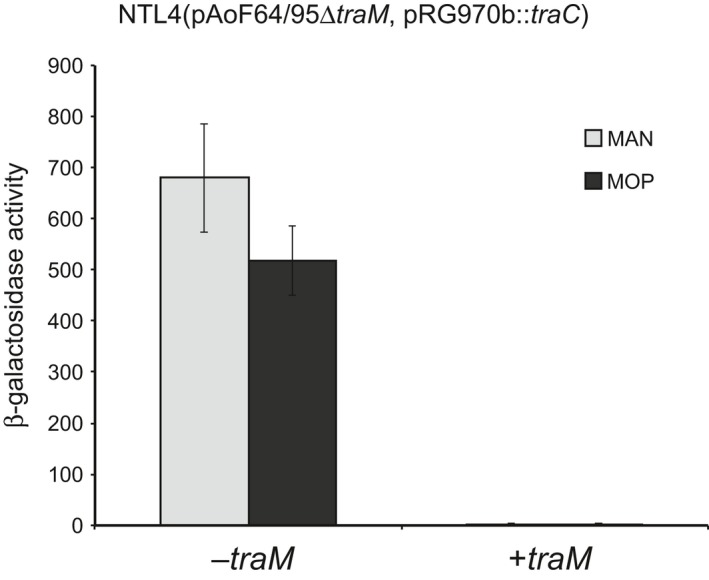
*traC* is expressed at high levels in the absence of TraM. Transcription of the *traC* promoter was assessed in both the presence and absence of TraM using a *traC* transcriptional fusion to *lacZ* on pRG970b::*traC*. This reporter plasmid was placed *in trans* to pAoF64/95Δ*traM* in strain NTL4 harboring either pSRKGm or pSRKGm::*traM*. Both strains were grown in AB minimal medium with mannitol (light gray bars) or mannopine (dark gray bars) as the primary source of carbon and supplemented with 1 mmol/L IPTG to induce expression of *traM*. Samples of each culture were collected and assessed for β‐galactosidase activity as described in Experimental Procedures. n = 3 and error bars represent the standard deviation

The *traM* mutant of pAoF64/95 is constitutive for transfer and produces high levels of AAI, even when grown in the absence of MOP (Wetzel et al., 2014). In the well‐studied systems, TraM functions as an antiactivator by interacting with TraR, thereby preventing the activator from binding its target promoter sites. We assessed the activity of TraM from pAoF64/95 by examining its effect on TraR‐mediated activation of the *traC* promoter as described in Experimental Procedures. We transformed the pRG970b*traC*::*lacZ* reporter plasmid into strain NTL4 harboring pAoF64/95Δ*traM* described previously (Wetzel et al., 2015) and then added either pSRKGm or pSRKGm::*traM*. We assessed these two strains for β‐galactosidase activity in cultures grown with either mannitol or MOP. In strains lacking *traM*, p*traC::lacZ* was expressed at high levels when the cells were grown with either carbon source (Figure 2). However, IPTG‐induced expression of the cloned *traM* reduced the β‐galactosidase activity to very low levels regardless of whether the cells were grown with mannitol or MOP (Figure 2).

### 3.3 Expression of TraR is not inducible by MOP

**Figure 3:**
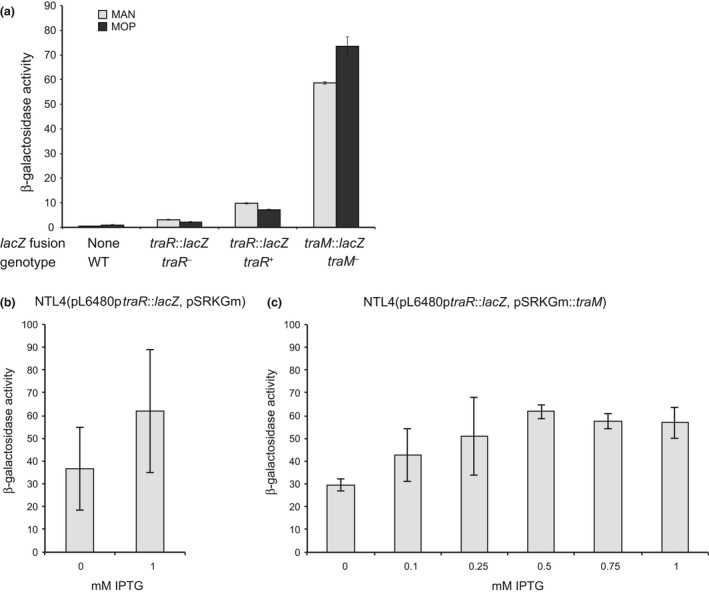
*traR* and *traM* are not significantly regulated at the level of transcription. Native expression of *traR* and *traM* was assessed utilizing *lacZ* transcriptional or translational fusions integrated into pAoF64/95. The translational fusions disrupt the target genes; the transcriptional fusion leaves an intact copy of the gene upstream of *lacZ*. Cells were subcultured into AB minimal medium with mannitol (light gray) or mannopine (dark gray) and grown to an approximate OD_600_ of 0.4 to 0.6. Cells were incubated, then harvested and assayed for β‐galactosidase activity as described in Experimental Procedures. n = 3 and error bars represent standard deviation. Expression of *traR* in both the presence and absence of TraM was assessed in strain NTL4 harboring pL6480*ptraR*::*lacZ*, a recombinant vector encoding *traR* translationally fused to *lacZ,* and either (b) pSRKGm (n = 2, standard deviation) or (c) pSRKGm::*traM* (n = 2, standard deviation). Cells were grown in ABM with increasing concentrations of IPTG to induce increasing amounts of TraM and assessed for expression of TraR as measured by β‐galactosidase activity as described in Experimental Procedures

In both nopaline and octopine‐type Ti plasmids transcriptional expression of *traR*, which is normally low, is induced in cells grown with the conjugative opine (Fuqua & Winans, 1994; Piper et al., 1999). We hypothesized that growth with mannopine would have a similar affect on the expression of *traR*_pAoF64/95_. To test this hypothesis, we created two separate *lacZ* fusions to *traR* in pAoF64/95 as described in Experimental Procedures. The first, a transcriptional fusion, yields in addition an intact copy of the *traR* gene. The second, a translational fusion, disrupts *traR*. We assessed β‐galactosidase activity in these two strains grown with either mannitol or MOP as the primary carbon source. In each case, levels of expression of *traR* were relatively low (Figure 3a). However, unlike other opine‐inducible plasmids, the expression of *traR* did not increase in response to growth with MOP in either reporter strain (Figure 3a).

### 3.4 TraM does not influence expression of *traR*

To assess if TraM directly regulates the expression of *traR*, we constructed a separate transcriptional fusion of the promoter of *traR* to *lacZ*. This recombinant reporter plasmid, pL6480p*traR*::*lacZ* (Table S1), was transformed into strain NTL4, along with pSRKGm::*traM*. The expression level of TraM in strains harboring this vector is dependent upon the concentration of IPTG. We assessed β‐galactosidase activity of p*traR*::*lacZ* in the reporter strains grown in minimal liquid medium supplemented with IPTG at concentrations ranging from 0 to 1 mmol/L to induce expression of TraM. In comparison to the control culture, expression of *traR* was not substantially affected by expression of *traM* (Figure 3b and c).

### 3.5 TraM is expressed constitutively at high levels and increases slightly in response to MOP

That *traR* of pAoF64/95 is expressed constitutively rules out a regulatory mechanism that involves increased expression of the activator titrating out the antiactivator. As an alternative, we considered the hypothesis that the expression of TraM would decline in cells grown with MOP, thereby allowing accumulation of active TraR. We utilized a previously constructed *traM*::*lacZ* translational fusion in pAoF64/95 (Wetzel et al., 2014) and assessed this strain for β‐galactosidase activity when cells were grown with mannitol or MOP as the primary carbon source. The reporter strain expressed relatively high levels of β‐galactosidase when cells were grown with mannitol and β‐galactosidase levels actually increased slightly when the cells were grown with MOP (Figure 3a). We conclude from these experiments that both TraR and TraM are expressed constitutively and that induction of conjugation following growth with MOP does not involve regulating transcription of either the activator or its antiactivator.

### 3.6 MrtR negatively regulates transfer of pAoF64/95

**Figure 4:**
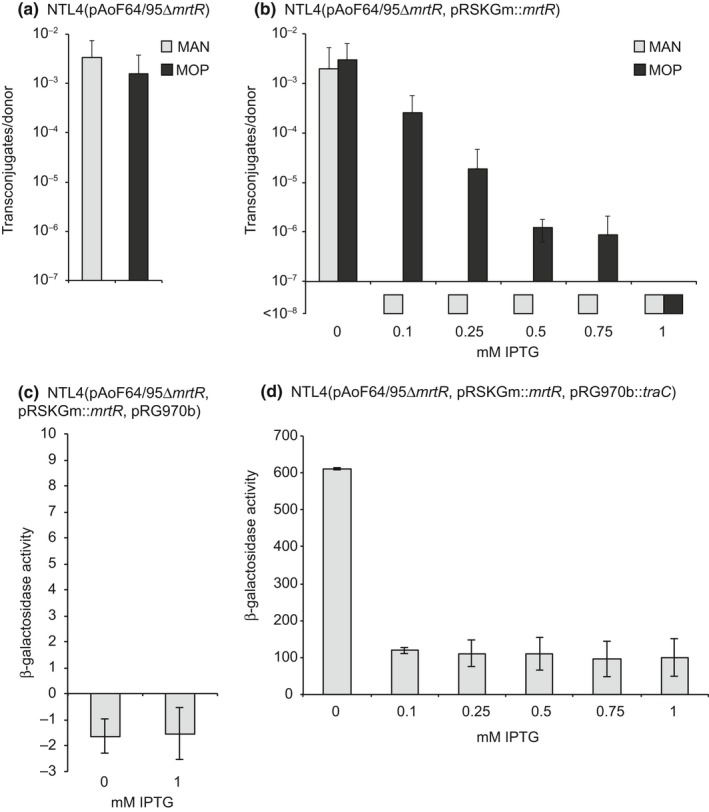
MrtR inhibits transfer of pAoF64/95 and expression of the tra regulon. The *mrtR* mutant, NTL4(pAoF64/95Δ*mrtR*) (a) and the mutant complemented with a cloned wild‐type copy of *mrtR* (b) were assessed for conjugative transfer when grown in AB minimal medium with mannitol (light gray) or mannopine (dark gray) as the primary source of carbon. The effect of MrtR on expression of the *tra* regulon was assessed in the complemented mutant, (NTL4[pAoF64/95Δ*mrtR*, pSRKGm::*mrtR*]), harboring (c) pRG970b, or (d) the *lacZ* reporter pRG970b::*traC*. Strains were grown with or without IPTG to induce expression of MrtR, and assayed for β‐galactosidase activity as described in Experimental Procedures. In all cases, n = 2 and error bars represent standard deviation, except in (b) where 0 and 1 mmol/L IPTG concentrations have an n = 3

Both TraR and TraM are expressed constitutively, yet transfer of pAoF64/95 is inducible when cells are grown with MOP. This set of observations suggests that another MOP‐dependent factor must be involved in allowing TraR levels to overcome antiactivation by TraM. Sequence analysis of pAoF64/95 revealed a potential regulator located within the MOP catabolism locus (Wetzel et al., 2014). This putative regulator is related to members of the GntR family of transcriptional regulators, a family of proteins that generally contain both a DNA‐binding domain and an effector‐binding or oligomerization domain. We constructed an in‐frame allele replacement mutant of the open reading frame of this gene (Wetzel et al., 2015) and assessed the mutant strain for conjugative transfer when cells were grown with the noninducing carbon source, mannitol, or with MOP. Donors harboring the mutated plasmid transferred the kanamycin resistance trait at high frequencies, even when grown with mannitol (Figure 4a). Considering its involvement in mannopine‐regulated transfer, we have named this gene *mrtR* (mannopine regulation of transfer).

We confirmed the validity of the mutation by complementation with a wild‐type copy of *mrtR* cloned in pSRKGm. In this vector, expression of *mrtR* is inducible by IPTG. We grew NTL4(pAoF64/95Δ*mrtR*, pSRKGm::*mrtR*) with either mannitol or MOP, and increasing concentrations of IPTG. Donors grown with mannitol and IPTG added to 0.1, 0.25, 0.5, 0.75, and 1 mmol/L exhibited transfer frequencies repressed to levels similar to a strain carrying the wild‐type plasmid (Figure 4b). Transfer was induced by growth with MOP in donor cultures incubated with IPTG at concentrations ranging from 0.1 to 0.75 mmol/L IPTG (Figure 4b). However, in donors grown with 1 mmol/L IPTG transfer was fully repressed, even when cells were grown with the opine (Figure 4b). We conclude from these results that MrtR in some way negatively regulates transfer of pAoF64/95 and that such repression is lifted when donors are grown with MOP.

### 3.7 MrtR negatively regulates the genes for conjugative transfer

Considering that the *mrtR* mutant is constitutive for transfer, the genes for conjugative transfer should also be expressed at high levels when the mutant is grown with mannitol. To test this presumption, we assessed the expression of a TraR‐dependent promoter, *ptraC*, in the background of the *mrtR* mutant by transforming pRG970b*traC*::*lacZ* into NTL4(pAoF64/95Δ*mrtR*). We further complemented this strain with the IPTG‐inducible *mrtR* expression plasmid, pSRKGm::*mrtR*. The *traC* reporter strain was assessed for β‐galactosidase activity when grown with mannitol and with or without IPTG. In the *mrtR* mutant background, expression of *traC* is high in the absence of IPTG (Figure 4d). When even low levels of IPTG were added, the expression of *traC* decreased by approximately fivefold indicating that MrtR either directly or indirectly represses at least the *traCDG* operon (Figure 4d).

### 3.8 MrtR levels increase slightly in response to MOP

**Figure 5:**
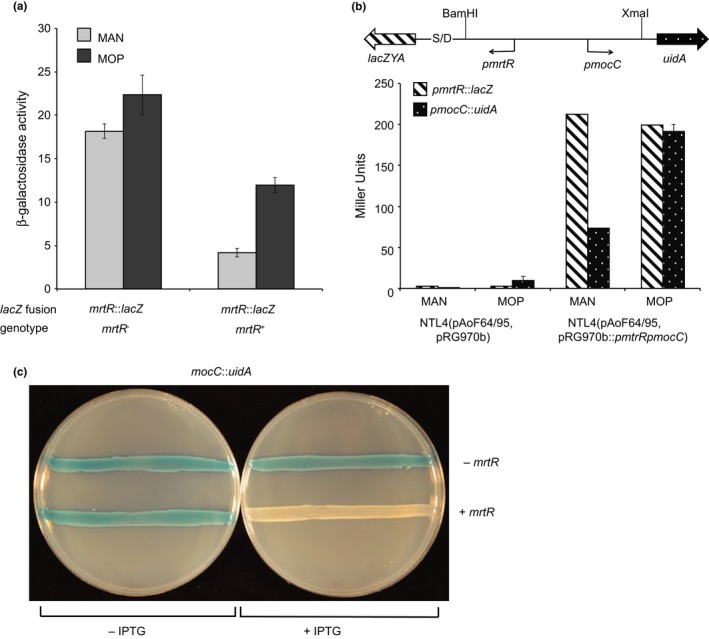
Expression of *mocC* is inducible by MOP and MrtR represses the *mocC* promoter. (a) The expression of *mrtR* was assessed using both transcriptional (n = 2, standard deviation) and translation (n = 3, standard deviation) *lacZ* fusions to the gene on pAoF64/95. Creation of the transcriptional fusion resulted in an intact copy of the target gene, while the translational fusion disrupted the gene of interest. Each strain was assessed for β‐galactosidase activity when grown in AB minimal medium with either mannitol (light gray) or mannopine (dark gray) as the primary source of carbon. Expression from the *mrtR* and *mocC* promoters was assessed using pRG970b::*pmrtRpmocC,* which contains the *mrtR*‐*mocC* intergenic region cloned into the bidirectional reporter plasmid pRG970b such that the *mocC* promoter is fused to *uidA* and the *mrtR* promoter is fused to *lacZ* (n = 1, error bars represent the standard deviation of the two internal replicates) (b). The strains harboring pAoF64/95 and this reporter plasmid were grown with either mannitol (MAN) or mannopine (MOP) and assessed for both β‐galactosidase and β‐glucoronidase activities as described in Experimental Procedures (b). The influence of MrtR on transcription of the *mocC* promoter was assessed using strain NTL4(pRG970b::*pmrtRpmocC*, pSRKGm::*mrtR*) and as a control NTL4(pRG970b::*pmrtRpmocC*, pSRKGm) (c). Strains were streaked on ABM medium containing X‐gluc with (right plate) or without (left plate) the addition of 0.1 mmol/L IPTG

To determine if expression of *mrtR* is itself controlled by MOP we created two separate *mrtR*::*lacZ* fusions, one transcriptional, the other translational, in pAoF64/95 as described in Experimental Procedures. The transcriptional fusion left an intact copy of *mrtR*, while the translational fusion disrupted the gene. We assessed each of these reporter fusion plasmids in strain NTL4 for β‐galactosidase activity when grown with either mannitol or MOP as the primary carbon source. In comparison to cells grown with mannitol, we observed a 1.2‐fold increase in β‐galactosidase activity when the translational fusion strain was grown with MOP and a 2.9‐fold increase in activity when the transcriptional fusion strain was grown with the opine (Figure 5a). MrtR induction by mannopine could be a mechanism to ensure that transcription of its target genes is repressed quickly once the opine signal is no longer present. Alternatively, MOP and concomitant activation of the QS system could increase the copy number of the plasmid as has been demonstrated in the Ti plasmid group as well as a plasmid harbored by *Rhizobium* (Li & Farrand, 2000; McAnulla, Edwards, Sanchez‐Contreras, Sawers, & Downie, 2007; Pappas & Winans, 2003).

### 3.9 TraR, TraM, MrtR, and AAI are not sufficient to induce transcription of the *traAFBH* promoter

**Figure 6:**
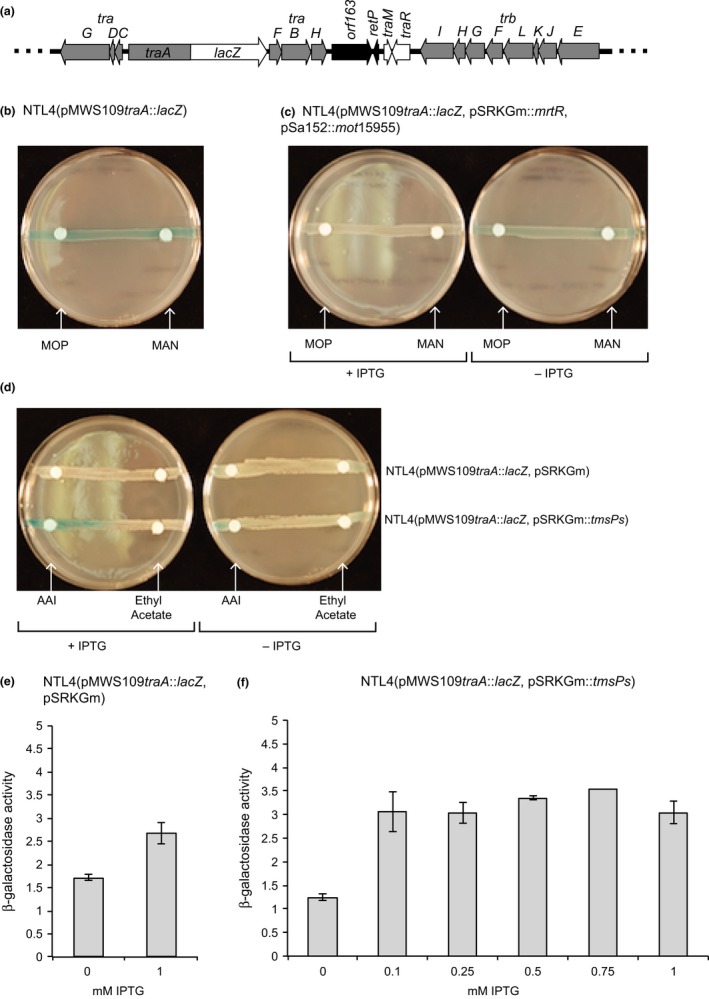
TmsP, but not MrtR, regulates a minimal plasmid system that contains TraR, TraM, and part of the *tra* regulon. (a) A map of the relevant region of pMWS109*traA*::*lacZ*, which contains the two *tra* operons, *traM*,* traR*, and the distal end of the *trb* operon. (b) Strain NTL4(pMWS109*traA*::*lacZ*) was assessed for β‐galactosidase activity on solid AB minimal medium supplemented to 0.05% with mannitol and containing X‐gal and AAI. Paper discs impregnated with either mannopine (MOP) or mannitol (MAN) were placed on top of each streak. (c) The influence of MrtR and mannopine on expression of *traA* was assessed by streaking a strain harboring the minimal reporter plasmid, an inducible source of *mrtR*, and the mannopine transporter on AB minimal medium supplemented to 0.05% with mannitol and containing X‐gal, AAI, and IPTG to induce expression of *mrtR* (left plate) and as a control, the same media lacking IPTG (right plate). The effect of TmsPs on transcription of *traA* in the minimal plasmid system was examined using NTL4(pMWS109*traA*::*lacZ*) harboring either pSRKGm::*tmsPs* or the empty vector, pSRKGm. The strains were tested qualitatively on solid AB minimal medium supplemented to 0.2% mannitol and containing X‐gal and either IPTG to induce expression of *tmsPs* (left plate) or, as a control, without IPTG (right plate) (d). Since *traI* is not encoded on pMWS109*traA*::*lacZ*, AAI was added either directly to the media as indicated or, AAI was added to Whatman discs that were placed on top of the bacterial streaks. When AAI was supplemented on Whatman discs, ethyl acetate was used as a control. (e and f) Expression of the *traA*::*lacZ* fusion in the minimal plasmid system in the two reporter strains was quantitated by measuring β‐galactosidase activity in cells grown in liquid cultures of AB minimal medium supplemented with AAI and with increasing amounts of IPTG to induce increasing amounts of TmsPs (n = 2, standard deviation)

If MrtR is negatively controlling expression of the *tra* regulon in response to MOP, we hypothesized that the *tra* genes on a cosmid that lacks *mrtR* would be expressed constitutively and that if MrtR was then added to the system the *tra* genes would be repressed. To test this hypothesis, we constructed a minimal plasmid system using a cosmid clone pMWS109 containing a *lacZ* fusion to *traA*. This cosmid encodes the two *tra* operons, *traM*,* traR* and part of the *trb* operon, but does not include *traI* through *trbD* (Figure 6a). We assessed expression of the *traA* reporter in strain NTL4(pAoF64/95) containing this cosmid *in trans* when the strain was grown on solid minimal medium supplemented with X‐gal. The reporter cosmid showed strong MOP‐dependent induction of β‐galactosidase activity (data not shown). We then tested the reporter cosmid in strain NTL4 in the absence of the megaplasmid on solid minimal medium containing mannitol and supplemented with AAI. No enhancement of β‐galactosidase activity was observed around paper discs containing either MOP or mannitol (Figure 6b). We additionally transformed pSRKGm::*mrtR* into NTL4(pMWS109*traA*::*lacZ*) along with a copy of the mannopine transporter from strain 15955 cloned into pSa152 to create NTL4(pMWS109*traA*::*lacZ*, pSRKGm::*mrtR*, pSa152::*mot*15955). We repeated the experiment adding IPTG to the media to induce expression of *mrtR*. Again, no increase in β‐galactosidase activity was observed when either MOP or mannitol was added to the paper discs (Figure 6c). These results suggest either that an essential factor is missing from our minimal system, or that MOP is not the true inducer molecule.

### 3.10 Expression of *mocC* is up‐regulated when cells are grown with MOP, and MrtR is likely the regulator of MOP catabolism and uptake

**Figure 7:**
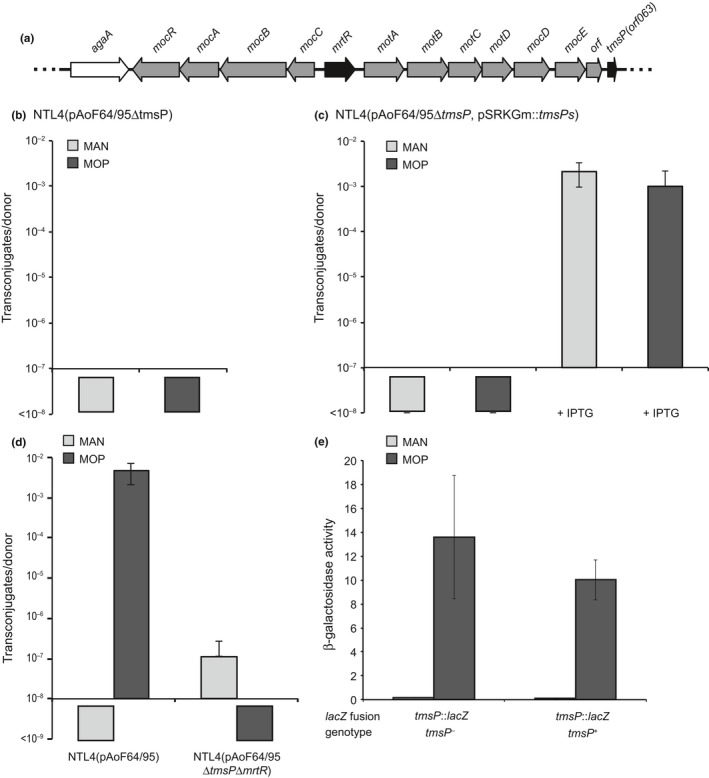
TmsP is essential for transfer, inducible by mannopine, and acts downstream of MrtR. (a) Organization of the genes on pAoF64/95 for uptake and catabolism of mannopine. The gene in white is a member of the agropinic acid uptake operon, the genes in black are the key regulators of mannopine catabolism and conjugative transfer and all other genes are gray. (b) Effect of the *tmsP* mutation on conjugative transfer of pAoF64/95Δ*tmsP* to C58C1RS was assessed following growth of the donor on minimal medium containing mannitol (light gray) or mannopine (MOP, dark gray) (n = 3). (c) The specificity of the *tmsP* mutation on pAoF64/95 was assessed by complementation with a cloned copy of *tmsPs*. The complemented strain was grown in minimal medium containing either mannitol (light gray) or mannopine (dark gray) and with either 0 or 1 mmol/L IPTG to induce expression of *tmsPs* (n = 2, standard deviation). (d) Epistasis between *tmsP* and *mrtR* was tested by assessing conjugative transfer of the double mutant plasmid, pAoF64/95Δ*tmsP*Δ*mrtR*, to C58C1RS when the donors were grown on either mannitol (light gray) or mannopine (dark gray) (n = 2, standard deviation). (e) Influence of growth with mannopine on expression of *tmsP* was assessed using both transcriptional and translation *lacZ* fusions to the gene on pAoF64/95. Creation of the transcriptional fusion resulted in an intact copy of *tmsP*, while the translational fusion disrupted the gene of interest. Each strain was assessed for β‐galactosidase activity following growth in medium containing either mannitol or mannopine as the primary source of carbon (n = 2, standard deviation)

The *mrtR* gene is adjacent and bidirectionally oriented to the operon encoding *mocC*, a gene essential for the catabolism of MOP (Figure 7a; Kim, Chilton, & Farrand, 1996; Kim & Farrand, 1996). This organization raises the possibility that MrtR regulates expression of the *mocCBAR* operon. To assess this, we cloned the intergenic region between *mrtR* and *mocC* into pRG970b such that the promoter for *mrtR* is transcriptionally fused to *lacZ* and the promoter for *mocC* is fused to *uidA* (Figure 5b). We transformed the resulting plasmid, pRG970b::*pmrtRpmocC*, or the empty vector pRG970b into strain NTL4(pAoF64/95), and the two reporter strains were grown in minimal medium supplemented with either mannitol or mannopine as the primary carbon source. β‐galactosidase and β‐glucuronidase activities were assessed using modified versions of the methods of Miller, (1972) and Jefferson (Jefferson et al., 1986). NTL4(pAoF64/95) harboring pRG970b expressed very low levels of both β‐galactosidase and β‐glucuronidase activity (Figure 5b). In cultures of NTL4(pAoF64/95) harboring the bidirectional reporter plasmid, expression corresponding to the *mrtR* promoter was relatively high in cells grown with either carbon source (Figure 5b). The *mocC* promoter expressed low levels of β‐glucoronidase activity when the reporter strain was grown with mannitol, but expression increased by almost threefold when the cells were grown with MOP (Figure 5b). These results indicate that the *mocCBAR* operon is inducible by MOP.

To assess if MrtR directly regulates *mocCBAR*, we transformed pRG970b::*pmrtRpmocC* into strain NTL4 and subsequently added either pSRKGm or pSRKGm::*mrtR*. These two reporter strains were grown without, or with the addition of IPTG at concentrations ranging from 0.1 to 1 mmol/L and β‐glucuronidase activity was assessed. The strain lacking *mrtR* expressed β‐glucoronidase activity when grown with or without IPTG (Figure 5c). The strain that harbored pSRKGm::*mrtR* demonstrated IPTG‐dependent repression of β‐glucoronidase activity, even when the lowest amount of IPTG was added (Figure 5c, and data not shown). These results indicate that MrtR represses the *mocC* promoter and therefore mannopine catabolism.

### 3.11 Another positive regulator of transfer

Given that MrtR regulates the *mocCBAR* operon, we considered the possibility that an unknown regulator of transfer is located somewhere in the *moc* regulon. We identified a small open reading frame (*orf063*, Wetzel et al., 2014) located ~500 nucleotides downstream of *mocE*, the last conserved gene in the MOP transport and catabolism operon (Figure 7a). The translated protein product of this orf aligns with a segment of the C‐terminal domain of other TraR proteins known to be involved in binding TraM (Figure S2). We assessed the influence of this locus by constructing an in‐frame kanamycin‐marked allele replacement mutant of this orf on pAoF64/95 as described in Experimental Procedures. The donor failed to transfer the mutated plasmid, even when grown with MOP (Figure 7b). We renamed this orf *tmsP* (*t*: tra; *m*: M; *s*: sequestering; *P*: protein), identified three potential translational start sites (Figure S3), and cloned each version into pSRKGm creating pSRKGm::*tmsP* *s* (short), pSRKGm:: *tmsP* *m* (medium), and pSRKGm:: *tmsP* *l* (long). We transformed each complementation vector into NTL4(pAoF64/95Δ*tmsP*) and tested each donor for ability to transfer the megaplasmid. In a set of trial matings, each of the three forms of *tmsP* complemented the *tmsP* mutation (data not shown). We chose the shortest version, *tmsPs*, to use for all further analysis. We assessed NTL4(pAoF64/95Δ*tmsP*, pSRKGm:: *tmsPs*) for conjugative transfer when grown with either mannitol or MOP as the primary carbon source, and with or without 1 mmol/L IPTG to induce expression of *tmsP*. No transfer was detected when the strains were grown without IPTG regardless of the carbon source (Figure 7c). However, when the donors were grown with IPTG, the plasmid transferred at high frequencies even when grown in the absence of the conjugative opine (Figure 7c). We conclude that *tmsPs* positively regulates transfer of pAoF64/95.

### 3.12 The expression of *tmsP* is inducible by MOP and repressed by MrtR

The results presented above suggest that expression of *tmsP* is regulated by MOP. To assess this possibility, we constructed a *tmsP*::*lacZ* translational fusion that disrupts the gene (NTL4[pAoF64/95Δ*tmsP*::*lacZ*]) and a *tmsP*::*lacZ* transcriptional fusion where *lacZ* is downstream of a wild‐type copy of the gene (NTL4[pAoF64/95 *tmsP*::*lacZ*]) all as described in Experimental Procedures. Each *tmsP* reporter strain was grown in minimal medium supplemented with either mannitol or MOP and the cells were assayed for β‐galactosidase activity. In comparison to the cells grown with mannitol, expression of *tmsP*::*lacZ* fusions increased between 90‐ and 100‐fold in cells grown with MOP (Figure 7e).

**Figure 8:**
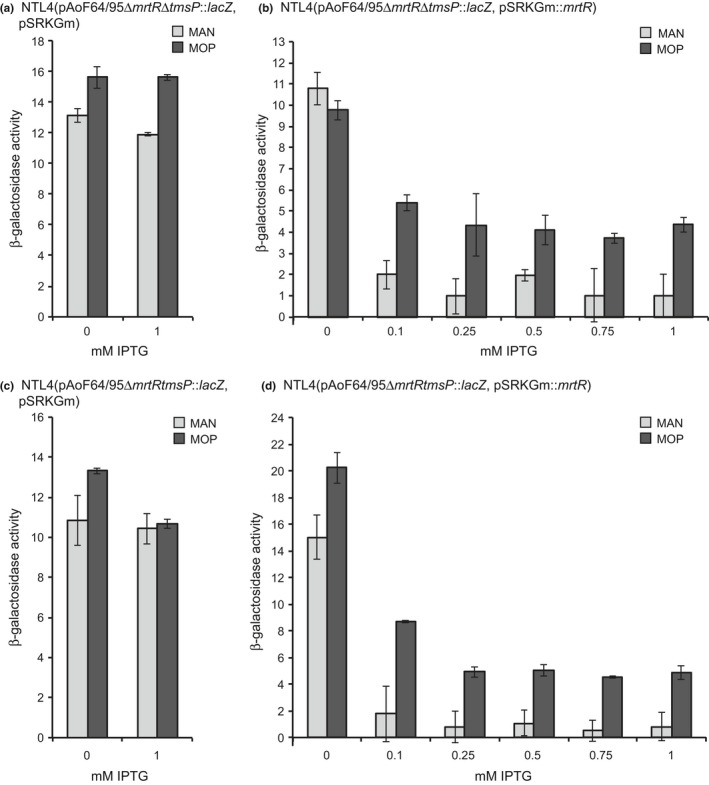
MrtR represses expression of *tmsP*. Expression of *tmsP* in both the presence (b and d) and absence (a and c) of *mrtR* cloned into pSRKGm was determined using the translational (a and b) and transcriptional (c and d) *lacZ* fusions to *tmsP* on pAoF64/95*mrtR*::*tetA*. All four strains were grown in minimal medium with mannitol or mannopine, without or with IPTG at concentrations ranging from 0 to 1 mmol/L as indicated. Strains were assessed for β‐galactosidase activity as described in Experimental Procedures. Each experiment was done twice with three internal repeats. The overall pattern of expression was similar between the two experiments. Only one experiment and the standard deviation of the three internal replicates is shown

We next determined whether MrtR regulates transcription of *tmsP*. We first constructed a *tetA*‐marked deletion derivative of *mrtR* in both NTL4(pAoF64/95Δ*tmsP*::*lacZ*) and NTL4(pAoF64/95*tmsP*::*lacZ*) and subsequently introduced an IPTG‐inducible copy of *mrtR* on pSRKGm to complement the mutant allele. Strains were grown in minimal medium containing either mannitol or MOP and with varying concentrations of IPTG. In the *mrtR* mutant, both the *tmsP*::*lacZ* transcriptional and translational fusions were expressed at high levels (Figure 8a and c). In the complemented mutants expression of the *tmsP*::*lacZ* reporter fusions were expressed at high levels in the absence of IPTG (Figure 8b and d). Addition of IPTG to induce expression of *mrtR* decreased *tmsP* expression from strain NTL4(pAoF64/95Δ*mrtR*Δ*tmsP*::*lacZ*, pSRKGm::*mrtR*) by up to 11‐fold when grown with mannitol and between 1.8‐ and 2.6‐fold when the same strain was grown with MOP (Figure 8b). Similarly, expression of *tmsP* from strain NTL4(pAoF64/95Δ*mrtRtmsP*::*lacZ*, pSRKGm::*mrtR*) decreased by up to 30‐fold when grown with mannitol and between two‐ and fourfold when grown with MOP (Figure 8d).

We further assessed the ability of NTL4 harboring a double Δ*tmsP*Δ*mrtR* mutant of pAoF64/95 to transfer to recipient C58C1RS. No transconjugates were detected indicating that *tmsP* is epistatic to *mrtR* (Figure 7d).

We modified our minimal component reporter system to reflect our observations that TmsP is a positive regulator of conjugative transfer, that MrtR negatively regulates *tmsP*, and that over‐expression of *tmsP* in the *tmsP* mutant not only complements the mutant, but does so even in the absence of MOP. We transformed either pSRKGm::*tmsP* or pSRKGm into the strain harboring the cosmid reporter, NTL4(pMWS109*traA*::*lacZ*) and grew these strains on minimal media with or without IPTG to induce expression of *tmsP*. In a semiquantitative experiment conducted on solid medium, β‐galactosidase activity was observed in the cells around a paper disc containing AAI, but only when IPTG was added to the medium (Figure 6d). We then grew strains NTL4(pMWS109*traA*::*lacZ*, pSRKGm) and NTL4(pMWS109*traA*::*lacZ*, pSRKGm::*tmsP*) in minimal medium supplemented with AAI and varying concentration of IPTG and assessed β‐galactosidase activity quantitatively. As compared to the control culture, expression of *traA*::*lacZ* from the cosmid increased up to threefold when IPTG was added to the culture (Figure 6f).

### 3.13 TmsP interacts with TraM

**Figure 9:**
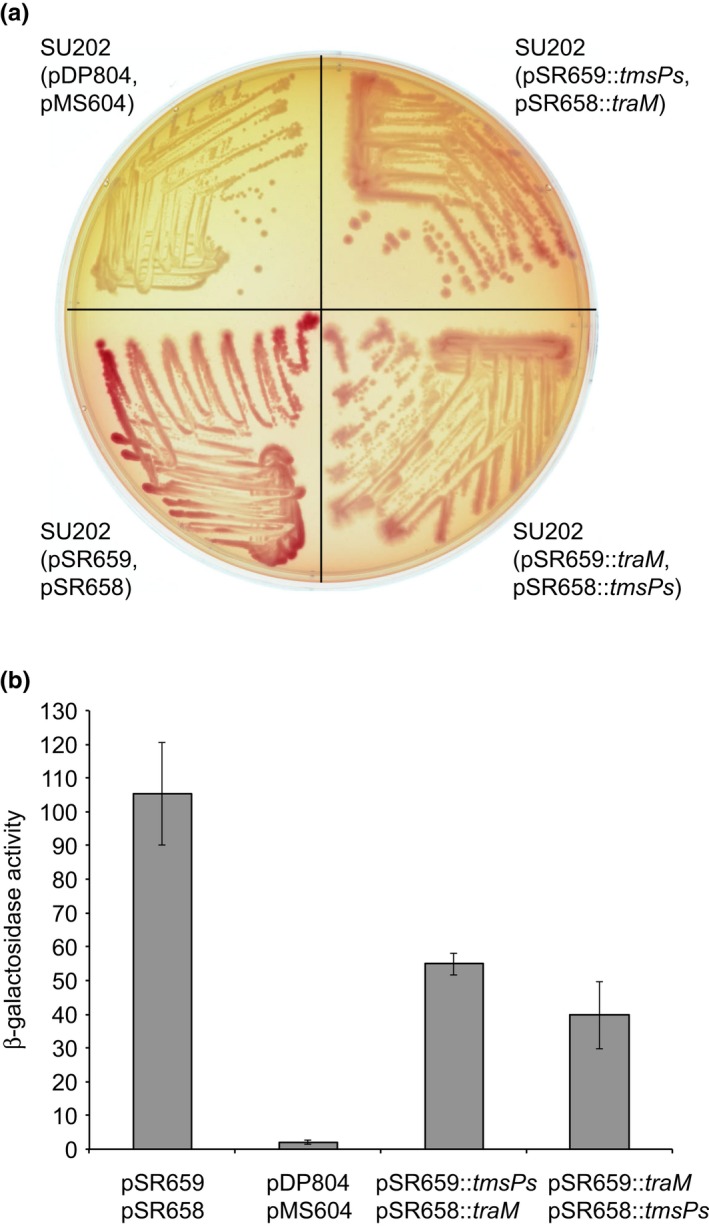
TmsPs interacts with TraM. Interaction between TmsPs and TraM was assessed using a bacterial two‐hybrid system as described in Experimental Procedures. *Escherichia coli* SU202 harboring empty vectors pSR659 and pSR658 acted as a negative control that expressed *lacZ*, while SU202(pDP804, pMS604), which has cloned copies of the *fos* and *jun* genes, encoding two proteins known to strongly interact and therefore repress expression of *lacZ*, served as a positive control. Plasmids pSR658 and pSR659 that carry inserts that express *traM* and *tmsPs* were transformed into SU202. Expression of β‐galactosidase was assessed both qualitatively using growth on McConkey plates (a) and quantitatively by assessing β‐galactosidase activity (b) (n = 3, standard deviation)

The short form of *tmsP* encodes a protein of 87 amino acids that shares between 30 and 38 percent identity and between 55 and 60 percent similarity with TraR_pTiC58_, TraR_pNGR234a_, and TraR_pAoF64/95_ (Figure S2). The region of relatedness overlaps the TraR‐TraM interaction interface (Figure S2; Chen, Jeffrey, Fuqua, Shi, & Chen, 2007; Hwang et al., 1999; Luo et al., 2000; Qin, Su, & Farrand, 2007). Considering the amino acid sequence similarity, that TraR and AAI are sufficient to activate the three promoters for transfer, and that a TraM mutant is constitutive for transfer, we hypothesized that the product of *tmsP* competes with TraR for interaction with TraM. To test this hypothesis, we utilized a bacterial two‐hybrid system where heterodimerization of the proteins of interest results in repression of *lacZ*. Colonies of the strain expressing the appropriate fusions of LexA to both TmsPs and TraM were lighter pink on MacConkey's medium as compared to those of the control strain harboring empty vectors pSR659 and pSR658 and darker in color in comparison to the white colonies of the positive control strain, Su202(pDP804, pMS604) (Figure 9a). Su202(pDP804, pMS604) expresses versions of LexA fused to Fos and Jun, two proteins known to strongly interact. In a quantitative analysis, the positive control strain exhibited 52‐fold lower levels of β‐galactosidase activity in comparison to the strain harboring the two empty vector plasmids. The strains co‐expressing the LexA fusions to TraM and TmsPs produced between two‐ and threefold lower levels of β‐galactosidase activity in comparison to the negative control strain (Figure 9b). These results are consistent with our hypothesis that the product of *tmsP*, interacts with TraM.

### 3.14 DFG, and not MOP, is the true inducer of conjugative transfer of pAoF64/95

**Figure 10:**
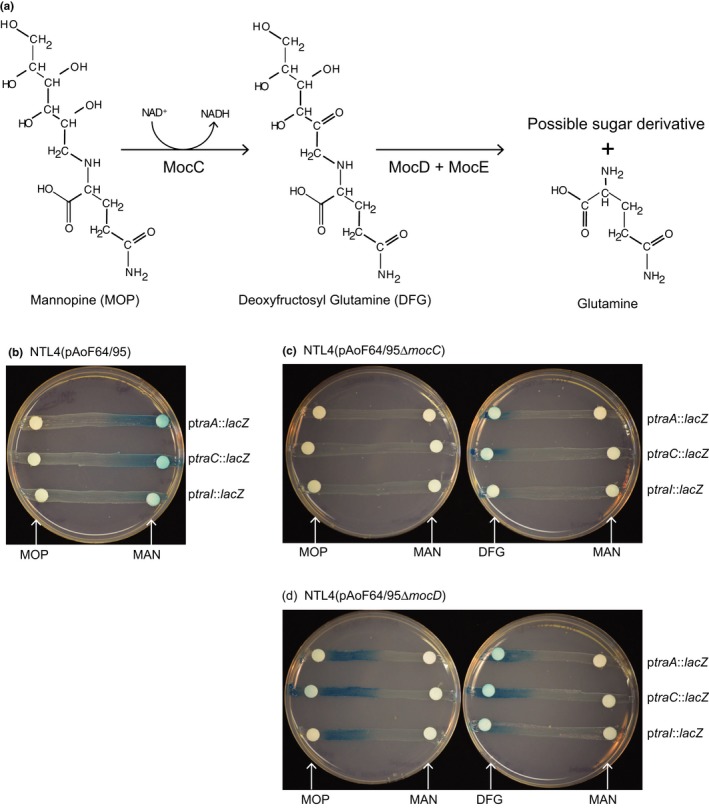
Deoxyfructosyl glutamine (DFG), and not mannopine (MOP), is the inducer of the *tra* regulon. (a) The pathway for catabolism of MOP (Kim & Farrand, 1996; Kim et al., 1996). (b–d) Activation of three TraR‐dependent promoters, *ptraA*,* ptraC*, and *ptraI*, was assessed using *lacZ* transcriptional fusions to the promoters of each gene cloned into pRG970b. Each reporter vector in a strain harboring wild‐type pAoF64/95 (b), a *mocC* mutant (c), or a *mocD* mutant (d) was assessed for β‐galactosidase activity qualitatively on solid ABM medium containing X‐gal. Discs impregnated with mannitol (MAN), MOP, or DFG were placed on the streaks all as described in Experimental Procedures

The initial experiments with the minimal plasmid system introduced the possibility that MOP is not the direct inducer of conjugative transfer. We hypothesized that deoxy‐fructosyl glutamine (DFG), the first intermediate of MOP catabolism (Kim & Farrand, 1996) could be the true effector molecule. We examined this question using two mutants of pAoF64/95, pAoF64/95Δ*mocC* and pAoF64/95Δ*mocD*. The *mocC* mutant is unable to convert MOP into DFG, but should be able to catabolize DFG, while the *mocD* mutant can convert MOP into DFG, but cannot further degrade this intermediate (Figure 10a). To assess expression of the transfer genes in the two mutants, we transformed three TraR‐dependent promoter reporter plasmids where *traA*,* traC*, or *traI* is transcriptionally fused to *lacZ* into strain NTL4 carrying either pAoF64/95, pAoF64/95Δ*mocC*, or pAoF64/95Δ*mocD*. We assessed these reporter strains for β‐galactosidase activity on solid minimal medium containing 0.05% mannitol and supplemented either with mannitol, MOP, or DFG on discs of Whatman paper as described in the Experimental Procedures. In strains harboring the wild‐type plasmid, MOP, but not mannitol induced activation of the three reporter strains (Figure 10b). The *mocC* mutant harboring the reporter fusions showed no TraR‐dependent activation when either MOP or mannitol was supplied, but was activated by DFG (Figure 10c). The *mocD* mutant containing the reporter plasmids exhibited β‐galactosidase activity when either MOP or DFG were present, but not when the alternate carbon source, mannitol, was supplied (Figure 10d). These results suggest that DFG, and not MOP, is the effector molecule for opine‐inducible conjugative transfer of pAoF64/95.

## 4 DISCUSSION

Quite clearly, growth with mannopine induces conjugative transfer of pAoF64/95 by activating the quorum‐sensing system composed of the QS activator TraR and TraI, the acyl‐HSL synthase. However, unlike what has been described in Ti plasmids and the opine‐catabolic plasmid pAtK84b, growth with the conjugative opine does not induce the transcriptional expression of *traR*. Nor does growth with mannopine repress expression of *traM*; both key QS regulatory genes are expressed constitutively.

**Figure 11:**
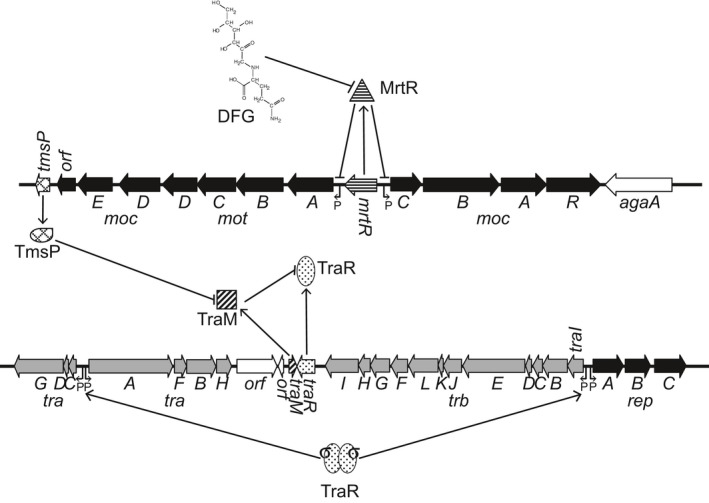
Proposed model of DFG‐inducible transfer of pAoF64/95. The genes involved in mannopine uptake and utilization are colored black, the *tra* regulon is light gray, and the plasmid replication and partitioning genes are dark gray. The genes and proteins involved in mannopine‐inducible transfer are as follows: *traR*/TraR, black dots on a white background; *traM*/TraM, diagonal stripes; *mrtR*/MrtR, horizontal stripes; and *tmsP*/TmsP, crosshatched. Agrobacterium autoinducer (AAI) is indicated by σ. All other genes are in white. Promoters are indicated by “P” and transcriptional direction is indicated by the arrows. Under noninducing conditions TraM binds to TraR preventing activation of the three promoters of the *tra* regulon while MrtR represses transcription of the mannopine catabolism and uptake operons including *tmsP*. When DFG is present, repression by MrtR is relieved and *tmsP* is transcribed. TmsP binds to TraM and sequesters the antiactivator thereby freeing TraR, which, as long as the population has reached its critical threshold and enough AAI is available, can form active dimers and activate transcription of the *tra* regulon

Growth with MOP apparently regulates the QS system via the small protein TmsP, a novel component that is homologous to a segment of the C‐terminal domain of TraR that interfaces with TraM. In other well‐studied systems, this interaction between TraM and TraR prevents inappropriate activation of the *tra* regulon (Hwang et al., 1999; Luo et al., 2000). We propose a model for regulation of transfer of pAoF64/95 in which in the absence of the conjugative opine, TraM is produced at a level that inhibits all of the constitutively produced TraR (Figure 11). When grown with the opine, TmsP is produced, binds to TraM, thereby reducing the amount of the antiactivator to a level in which it does not fully sequester the available TraR (Figure 11). Five lines of evidence are consistent with this model. First, *tmsP* mutants fail to transfer when grown with MOP. Thus, TmsP functions as a positive regulator of transfer. Second, transcription of *tmsP*, which is normally repressed, is strongly induced when the cells are grown with MOP. Third, mutants derepressed for expression of *tmsP* are constitutive for transfer. Fourth, results from a bacterial two‐hybrid analysis are consistent with our hypothesis that TmsP can interact with TraM. Finally, in support of our hypothesis, TmsP shows strong sequence similarity with several alleles of TraR in the region experimentally defined as important for interaction with TraM. Six of the eight residues of TraR from pTiC58 genetically and biochemically identified as being important in this interaction (Luo et al., 2000; Qin et al., 2007) are identical or strongly conserved in TmsPs (Figure S2). Similarly, of the four residues of TraR from pNGR234 identified by structural analysis as important for interaction with TraM (Chen et al., 2007) three are identical or conserved replacements in TmsPs (Figure S2). We considered the possibility that TmsP positively regulates transfer via a mechanism that is dependent on mannopine but independent of the TraR QS system. However, the fact that a *traR* mutant fails to transfer even when grown with MOP (Wetzel et al., 2014) argues against this model.

The *tmsP* open reading frame has three potential in‐frame translational start sites generating proteins of 113, 94, and 87 residues (Figure S3). The long and medium open reading frames (orfs) initiate from non‐canonical TTG start codons, while the short orf initiates from an ATG codon. Clones expressing each of these orfs from vector‐provided signals fully complemented the *tmsP* mutant suggesting that the smallest protein is functional. Moreover, expression of a fusion of the short orf to *lexA* gave a positive result in the bacterial two‐hybrid analysis.

*tmsP* is located just downstream from the last gene of the *motABCDmocDE* operon responsible for uptake and catabolism of mannopine (Wetzel et al., 2014). Whether the gene is part of this operon remains to be determined. However, our studies with *lacZ* fusions indicate that expression of *tmsP* responds to mannopine thereby providing a mechanism for linking regulation of the QS system to the appropriate exogenous opine signal. Remarkably, homologs of *tmsP* are distributed among a wide variety of plasmids with Class I QS‐regulated conjugative transfer systems found in members of the Rhizobiales. Most notably, in pRi1724 the *tmsP* homolog *riorf44* is located just downstream of a regulon described as possibly involved in opine catabolism (Moriguchi et al., 2001). This region of the Ri plasmid does not share any other sequences in common with pAoF64/95. Although pRi1724 is said to be conjugative (Tanaka, Matsumoto, & Machida, 1993), there have been no reports on whether transfer is regulated and if so, how it is controlled.

While both *tmsP* and *trlR* occupy similar locations just distal to the *moc* operons of pAoF64/95 and pTiR10/pTi15955, respectively, three lines of evidence suggest that the two genes do not share a common origin. First, the *moc* operons of the two plasmids are not entirely syntenic, especially at the junction between the *moc* genes and *tmsP*/*trlR* (Wetzel et al., 2014). Second, while *tmsP* represents only a small fragment of *traR*,* trlR* spans the entire *traR* gene but contains a single bp deletion resulting in a translational frameshift (Oger et al., 1998; Zhu & Winans, 1998). Third, sequence relatedness analyses indicate the amino acid sequences of TmsP and the repaired version of TrlR are only distantly related. We consider it more likely that *tmsP* is a fragment of some other *traR*‐like gene. Such fragments are scattered throughout many RepABC plasmids in the Rhizobiales. These observations suggest that the association of *tmsP* and *trlR* with the *moc* regulon arose by fortuitous but independent fusion events during the evolution of these plasmids.

Our results indicate that the opine signal is transmitted to the QS system through the novel negative regulator, MrtR. This repressor contains an N‐terminal GntR‐like winged helix‐turn‐helix DNA‐binding domain and a C‐terminal UTRA family ligand‐binding domain. Given that a *mrtR* mutant expresses *tmsP* constitutively, the repressor clearly regulates this gene. Whether it also regulates the entire *motABCDmocDE* operon remains to be determined. However, the promoter regions of both *moc* operons contain an identical 14 bp perfect inverted repeat (Figure S4A). In turn, this repeat is virtually identical to the 12 bp consensus operator sequence, differing only by a TA basepair at the axis of dyad symmetry, that is recognized by ExuR, a GntR‐like repressor of *E. coli* (Figure S4B; Rigali, Derouaux, Giannotta, & Dusart, 2002). The *mrtR* gene is not present in the otherwise highly conserved *moc* regulon of the octopine‐ and succinamopine‐type Ti plasmids pTiR10/pTi15955 and pTiBo542 (Kim & Farrand, 1996; Oger & Farrand, 2002; Oger et al., 1998). Regulation of the *moc* regulon of pTi15955 is complex, involving two other repressors, MocR and MocS (Jung, Baek, Lee, & Kim, 1999). While the *moc* regulon of pAoF64/95 contains an ortholog of *mocR*, a *mocR* mutant of this plasmid neither overproduced AAI nor was constitutive for conjugative transfer, indicating that this regulator does not control the QS system (Figure S5).

Remarkably, MOP is not the true signal that activates the QS system of pAoF64/95. Rather deoxy‐fructosyl glutamine, the first intermediate in the catabolism of mannopine induces the system. That a metabolic intermediate is the true inducer is uncommon but not unique. For example, allolactose, a metabolic product of lactose is the true ligand for LacI (Burstein, Cohn, Kepes, & Monod, 1965; Jobe & Bourgeois, 1972), while tyramine, a catabolic intermediate in the conversion of phenylethylamine to phenylacetate, is the ligand recognized by FeaR, the AraC‐like activator that regulates the pathway in *Escherichia coli* (Zeng & Spiro, 2013). More relevant to this system, arabinose‐2‐phosphate, the first intermediate in catabolism of agrocinopines A and B, is the ligand recognized and bound by AccR, the FucR‐like repressor that controls expression of the *acc* operon as well as *traR* in the *arc* operon of pTiC58 (El Sahili et al., 2015).

RepABC plasmids with Class I QS‐regulated conjugative transfer systems fall into two organizational types, Group I and Group II (Wetzel et al., 2015). Remarkably, pAoF64/95 is the only Group II agrobacterial plasmid conclusively demonstrated to be self‐transmissible. The conjugative Ti plasmids described to date all show Group I organization, and the functional *traR* gene, which is contiguous with the *traAFBH* operon, is, in all cases, a member of a gene set the expression of which is controlled by the conjugative opine (Fuqua & Winans, 1994; Oger & Farrand, 2001; Oger & Farrand, 2002; Piper et al., 1999). This organization accounts for how the conjugative opine induces the QS system. On the other hand, while *traR* of pAoF64/95 is located at its canonical site just distal to *traAFBH*, the gene is monocistronic. Moreover, the structural and regulatory genes encoding catabolism of the conjugative opine are located some 70 kb from *traR*,* traM*, and the three operons of the *tra* regulon.

Opine‐mediated regulation of the activity of TraR of pAoF64/95 mediated by the putative anti‐antiactivator TmsP is novel and represents the third mechanism known by which exogenous signals activate the Class I conjugative transfer systems of rhizobial plasmids. In the first case, among the Ti plasmids examined to date, the signal, the conjugative opine, directly regulates transcription of *traR*. Control of transfer of pAoF64/95 is similar in that it responds to opines, but differs in that regulation of TraR activity is not at the level of transcription. In both cases the regulatory signal ties conjugative transfer to the environment of the crown gall tumors. In the second mechanism, transcription of *traR* on the rhizobial Sym plasmid pRL1JI is dependent upon a second LuxR homolog, encoded by *bisR* located in the *traR*‐*traM* cluster (Danino, Wilkinson, Edwards, & Downie, 2003; Wilkinson, Danino, Wisniewski‐Dye, Lithgow, & Downie, 2002). BisR, in turn, requires an acyl‐homoserine lactone signal produced by nearby strains of *Rhizobium* that lack the Sym plasmid, thus tying plasmid transfer to the availability of a suitable recipient (Danino et al., 2003; Lithgow et al., 2000; Wilkinson et al., 2002). In the case of pRL1JI, like the Ti plasmids, the regulatory mechanism involves increasing expression of *traR* to titrate TraM (Danino et al., 2003). In contrast, expression of *traR*_pAoF64/95_ is constitutive and TmsP titrates TraM posttranslationally. There may be additional regulatory mechanisms, but what is remarkable is that all three known strategies have in common the linkage of conjugative transfer to some environmental component. It is quite clear that it is important to these plasmids, and to the genetics that they confer on their host, to confine their transfer to well‐defined environmental conditions and niches of habitation.

## CONFLICT OF INTEREST

The authors have no conflicts of interest.
