# Supplementary material for: Quorum‐dependent transfer of the opine‐catabolic plasmid pAoF64/95 is regulated by a novel mechanism involving inhibition of the TraR antiactivator TraM
Source: Microbiologyopen. 2018 Apr 10;8(1):e00625. doi: 10.1002/mbo3.625 (PMC6341043; doi:10.1002/mbo3.625)

Supplemental Table 1. Bacterial strains and plasmids

| Strain or Plasmid | Relevant Characteristics[a] | Source or Reference |
|---|---|---|
| ***Agrobacterium spp.*** | | |
| C58C1RS | $Sm^r$, $Rif^r$; Ti-plasmidless derivative of C58; pAtC58 | Our collection |
| NTL4 | Ti-plasmidless derivative of C58; pAtC58; ΔtetAR | (Luo *et al.*, 2001) |
| ***Escherichia coli*** | | |
| DH5α | *λ*$^-$ ϕ80d*lacZ*ΔM15 Δ(*lacZYA-argF*) U169 *recA1 endA1 hsdR17*($r_k^-$, $m_k^-$) *supE44 thi-1 gyrA relA1* | Invitrogen |
| S17-1 | $Tp^r$, $Sm^r$, *recA*, *thi*, *pro*, *hsdR-M*+ RP4::2-Tc::Mu::Km Tn7 λpir | (Simon *et al.*, 1983) |
| S17-1/λpir | λpir lysogen of S17-1 | (Simon *et al.*, 1983) |
| Su202 | $Cm^r$, $Kan^r$; *lexA71*::Tn5(Def) *sulA211* *Δ(lacIPOZYA)169/F' lacI*$^q$*lacZΔM15*::Tn9 | (Dmitrova *et al.*, 1998) |
| **Plasmids** | | |
| pAoF64/95 | Wild type mannopine catabolism plasmid | (Wetzel *et al.*, 2014) |
| pAoF64/95::*mrtR*pVik107 | $Kan^r$; *mrtR*::*lacZ* translational fusion; Δ*mrtR* | This work |
| pAoF64/95::*mrtR*pVik112 | *Kan*$^r$*; mrtR*::*lacZ* transcriptional fusion; *mrtR*$^+$ | This work |
| pAoF64/95::*tmsP*pVik107 | *Kan*$^r$*; tmsP*::*lacZ* translational fusion; Δ*tmsP* | This work |
| pAoF64/95::*tmsP*pVik112 | *Kan*$^r$*; tmsP*::*lacZ* transcriptional fusion, *tmsP*$^+$ | This work |
| pAoF64/95::*traM*pVik107 | *Kan*$^r$*; traM*::*lacZ* translational fusion; Δ*traM* | (Wetzel *et al.*, 2014) |
| pAoF64/95::*traR*pVik107 | *Kan*$^r$*; traR*::*lacZ* translational fusion; Δ*traR* | This work |
| pAoF64/95::*traR*pVik112 | $Kan^r$; *traR*::*lacZ* transcriptional fusion; $traR^+$ | This work |
| pAoF64/95Δ*mocC* | $Kan^r$; Δ*mocC* | This work |
| pAoF64/95Δ*mocD* | $Kan^r$; Δ*mocD* | This work |
| pAoF64/95Δ*mrtR* | $Kan^r$; Δ*mrtR* | (Wetzel *et al.*, 2015) |
| pAoF64/95Δ*mrtRtmsP*::*lacZ* | $Kan^r$, $Tet^r$; Δ*mrtR*::*tetA* mutation of pAoF64/95::*tmsP*pVik112 | This work |
| pAoF64/95ΔmtrRΔ*tmsP*::*lacZ* | $Kan^r$, $Tet^r$; Δ*mrtR*::*tetA* mutation of pAoF64/95::*tmsP*pVik107 | This work |
| pAoF64/95Δ*tmsP* | $Kan^r$; Δ*tmsP* | This work |
| pAoF64/95Δ*tmsP*Δ*mrtR* | $Kan^r$, $Tet^r$; Δ*mrtR*::*tetA* mutation of pAoF64/95Δ*tmsP* | This work |
| pAoF64/95Δ*traM* | $Kan^r$; Δ*traM* | (Wetzel *et al.*, 2015) |
| pAoF64/95Δ*traR* | $Kan^r$; Δ*traR* | (Wetzel *et al.*, 2014) |
| pAW19 | $Amp^r$, $Kan^r$; *sacB* | (Metcalf *et al.*, 1996) |
| pDP804 | $Amp^r$; Expression plasmid carrying a *LexA*$_{1\text{-}87}$*408::Jun* | (Dmitrova *et al.*, 1998) |
| pJW106 | $Sp^r/Sm^r$; λ Red helper plasmid | (Quick *et al.*, 2010) |
| pKD4 | $Amp^r$; Frt flanked $Kan^r$ cassette | (Datsenko & Wanner, 2000) |
| pKD46 | $Amp^r$; λ Red helper plasmid | (Datsenko & Wanner, 2000) |
| pL6480p*traR*::*lacZ* | $Tet^r$, *traR*::lacZY of pLKC480::*traR* subcloned into pLAFR6 | This work |
| pLAFR6 | $Tet^r$; IncP; multilinker of pUC18 flanked by synthetic *trp* terminators | (Huynh *et al.*, 1989) |
| pLKC480 | $Kan^r$; plasmid carrying *lacZY* cassette | (Tiedeman & Smith, 1988) |
| pLKC480::*traR* | $Kan^r$; 5' intergenic region and 5' portion of *traR* cloned into pLKC480 | This work |

| | | |
|---|---|---|
| pMS604 | Tet$^r$; Expression plasmid carrying a *LexA*$_{1-87}$WT::*Fos* | (Dmitrova *et al.*, 1998) |
| pMWS109 | Tet$^r$; Cosmid clone of pAoF64/94 containing the *tra* operons through part of the *trb* operon | (Wetzel *et al.*, 2014) |
| pMWS109*traA*::*lacZ* | Tet$^r$, Amp$^r$/Cb$^r$; *traA*::*lacZ*; Tn3-HoHo1 transposed into pMWS109 | This work |
| pMWS112 | Tet$^r$; Cosmid clone of pAoF64/9 containing mannopine uptake and utilization genes, *mrtR, tmsP* | (Wetzel *et al.*, 2014) |
| pRG970::*traC* | Sp$^r$, Amp$^r$/Cb$^r$; *traC-traA* intergenic region of pAoF64/95 cloned into pRG970b; *traC*::*lacZ* | This work |
| pRG970b | Sp$^r$, Amp$^r$/Cb$^r$; Bidirectionall *lacZY* and *uidA* fusion vector | (Van den Eede *et al.*, 1992) |
| pRG970b::*pmrtRpmocC* | Sp$^r$, Amp$^r$/Cb$^r$; *mrtR-mocC* intergenic region cloned into pRG970b; mrtR::*lacZ; mocC*::*uidA* | This work |
| pRG970b::*traA* | Sp$^r$, Amp$^r$/Cb$^r$; *traA-traC* intergenic region cloned into pRG970b; *traA*::*lacZ* | (Wetzel *et al.*, 2015) |
| pRG970b::*traI* | Sp$^r$, Amp$^r$/Cb$^r$; *traI-repA* intergenic region cloned into pRG970b; *traI*::*lacZ* | This work |
| pSa152 | Sp$^r$, Kan$^r$, Cm$^r$; IncW cloning vector | (Tait *et al.*, 1983) |
| pSa152::*mot*15955 | Sp$^r$, Kan$^r$; mannopine transport region (*mot*) of 15955 cloned into pSa152 | This work |
| pSR658 | Tet$^r$; ColE1 expression plasmid for LexA-WT dimerization system | (Daines *et al.*, 2002) |
| pSR658::*tmsPs* | *lexA*-WT::*tmsPs* | This work |
| pSR658::*traM* | *lexA*-WT::*traM* | This work |
| pSR659 | Amp$^r$; p15A expression plasmid for LexA-MUT dimerization system | (Daines *et al.*, 2002) |
| pSR659::*tmsPs* | *lexA*-MUT::*tmsPs* | This work |
| pSR659::*traM* | *lexA*-MUT::*traM* | This work |
| pSRKGm | Gm$^r$; IPTG-inducible expression vector | (Khan *et al.*, 2008) |
| pSRKGm::*mrtR* | Gm$^r$; *mrtR* cloned into pSRKGm | This work |
| pSRKGm::*tmsPl* | Gm$^r$; longest version of *tmsP* cloned into pSRKGm | This work |
| pSRKGm::*tmsPm* | Gm$^r$; medium version of *tmsP* cloned into pSRKGm | This work |
| pSRKGm::*tmsPs* | Gm$^r$; shortest version of *tmsP* cloned into pSRKGm | This work |
| pSRKGm::*traM* | Gm$^r$; *traM* cloned into pSRKGm | (Wetzel *et al.*, 2014) |
| pSRKGm::*traR* | Gm$^r$; *traR* cloned into pSRKGm | (Wetzel *et al.*, 2014) |
| pSShe | Cm$^r$; transposase helper plasmid, *tnpA*$^+$ | (Stachel *et al.*, 1985) |
| pTnHoHo1 | Amp$^r$/Cb$^r$; Tn3-HoHo1, transposable *lacZYA* sequence, *tnpA*$^-$, *tnpR*$^+$ | (Stachel *et al.*, 1985) |
| pVik107 | Kan$^r$; promoterless *lacZY*; *pir* dependent; used for translational fusions | (Kalogeraki & Winans, 1997) |
| pVik112 | Kan$^r$; promoterless *lacZY*; *pir* dependent; used for transcriptional fusions | (Kalogeraki & Winans, 1997) |
| pWM91 | Amp$^r$; *sacB* | (Metcalf *et al.*, 1996) |

[a] Sm$^r$, streptomycin resistance; Rif$^r$, rifampicin resistance; Tp$^r$, trimethoprim resistance; Cm$^r$, chloramphenicol resistance; Kan$^r$, kanamycin resistance; Tet$^r$, tetracycline resistance; Sp$^r$, spectinomycin resistance; Amp$^r$, ampicillin resistance; Cb$^r$, carbenecillin resistance; Gm$^r$, gentamicin resistance.

Supplemental Table 2. PCR primers used and their purposes

| Plasmid | Purpose of resulting construct | Primer names | Primer sequences or citation |
|---|---|---|---|
| pRG970b::*traC*$_{\text{pAoF64/95}}$ | *ptraC*::*lacZ* | traClacZBamHI | GCTAGAGGATCCTGGGTGAGAAAGCTTGTC |
| | | traAuidAXmaI | GCTAGACCCGGGAAATCTTTTCCCGGTTGC |
| pRG970b::*traI*$_{\text{pAoF64/95}}$ | *ptraI*::*lacZ* | traIlacZBamHI | GGTATAGGATCCTGTGGGTCGGCGGGGAAG |
| | | repAuidAXmaI | GGTATACCCGGGGCAAACTATATCCTGGGAGGCTAAAC |
| pRG970b::*mrtRmocC* | *pmocC*::*uidA* | mocCuidAXmaI | GCTAGACCCGGGTCTTCACTTTCTAACGAAGC |
| | | mrtRlacZBamHI | GCTAGAGGATCCCAATTGGTATGCTTGC |
| pLKC480::*traR* | *traR*::*lacZ* | traRintersenseSalI | GAATTCTATGTCGACAGAGCTATTGC |
| | | traRinterantisenseHindIII | TTTGATCATCCGTTCGTCATGCGAAGCTTCTGTCATGTCG |
| pVik112::3'*traR* | *traR*::*lacZ* | midtraRpVIk107EcoRI | GAGTACGAATTCCCGCATCGACCCGGTG |
| | | midtraRpVIk107SalI | GAGTACGTCGACGGGTATAGTCACCCCAC |
| pVik107::mid*traR* | Δ*traR*::*lacZ* | 3'traRpVik112EcoRI | GTCGACGAATTCGGTTCTGGCAATCCATTAC |
| | | 3'traRpVik112KpnI | GGATCCGGTACCCTACAGCAGGCCGTGGTC |
| pVik112::3'*mrtR* | *mrtR*::*lacZ* | 3'mrtRpVik112EcoRI | CAGCTGGAATTCTGAATCTCTCAGCGGGCGCT |
| | | 3'mrtRpVik112KpnI | CAGCTGGGTACCTAGCCACCTTTGTGTGGTGT |
| pVik107::mid*mrtR* | Δ*mrtR*::*lacZ* | midmrtRpVik107XmaI | CAGCTGCCCGGGCAAGATCTCCTGCGGTCTATT |
| | | midmrtRpVik107SalI | CAGCTGGTCGACTTCACTTATGGGCCGGTCGAA |
| pVik112::3'*tmsP* | *tmsP*::*lacZ* | 3'tmsPEcoRIpVik112 | GCATGACTGAATTCCGGTGGATTTCTCAAGGCAGG |
| | | 3'tmsPXbaIpVik112 | GCATGACTTCTAGATTATTCAACCGGCTTTTGAAGCTC |
| pVik107::mid*tmsP* | Δ*tmsP*::*lacZ* | midtsmPEcoRIpVik107 | GCATGACTGAATTCGAGTGCGAGTTTGTCATGAAG |
| | | midtmsPSalIpVik107 | GCATGACTGTCGACCGCGCGCTCAAGGCAGAGCTC |
| pSa152::*mot*19555 | Expression of the mannopine transporter | motA15955XmaI | GGTATACCCGGGACGACGCTGATTTCGCA |
| | | motD15955XmaI | GGTATACCCGGGTCGTTTTCCCCTCTAGGGG |
| pSRKGm::*mrtR* | Expression of *mrtR* | mrtRsenseNdeI | GCCCGAGCTCATATGGACACCGAGCCAATGTAC |
| | | mrtRantisenseBamHI | GCGGAGCTCGGATCCTAGCCACCTTTGTGTGGTG |
| pSRKGm::*tmsPl* | Expression of *tmsPl* | tmsPstart1NdeI | CTAGCTAGCATATGGCTGCCGTCGCACAG |
| | | tmsPstopBamHI | CTAGCTAGGGATCCTTATTCAACCGGCTTTTGAAGC |
| pSRKGm::*tmsPm* | Expression of *tmsPm* | tmsPstart2NdeI | CTAGCTAGCATATGGCTTCAACCGACACTTCAATGC |
| | | tmsPstopBamHI | See above |
| pSRKGm::*tmsPs* | Expression of *tmsPs* | tmsPstart3NdeI | CTAGCTAGCATATGCTGGCGTCCGGTACCAA |
| | | tmsPstopBamHI | See above |
| pSR658::*traM* | Expression of *lexA*(WT)::*traM* | traMstartATGSacI | TCGATCGAGCTCATGAGCGACGTGAACTCG |
| | | traMendKpnI | TCGATCGGTACCTCAATCACCGACTTCGGG |
| pSR659::*traM* | Expression of *lexA*(Mut)::*traM* | traMstartATGSacI | See above |
| | | traMendKpnI | See above |
| pSR658::*tmsPs* | Expression of *lexA*(WT)::*tmsPs* | tmsPstart3XhoI | TCGATCCTCGAGATGCTGGCGTCCGGTAC |
| | | tmsPendPstI | TCGATCCTGCAGTTATTCAACCGGCTTTTGAAGCTC |
| pSR659::*tmsPs* | Expression of *lexA*(Mut)::*tmsPs* | tmsPstart3XhoI | See above |
| | | tmsPendHindIII | TCGATCAAGCTTATTCAACCGGCTTTTGAAGCTC |
| pMWS112Δ*mocC* | Δ*mocC* | mocCforwardKan | CTCGGGCGACAACTGCCCGCAGACAGCTTCGTTAGAAAGTGAAGAACCTGTGTAGGCTGGAGCTGCTTCG |
| | | mocCreverseKan | CAGACTTGCGCACAGACATCTAAATGCTCCCACGGCACCCATTGATCACATATGAATATCCTCCTTAGT |
| pMWS112Δ*mocD* | Δ*mocD* | mocDforwardKan | AAACCTGAAGCTTATACCCAACGCCTGACTAGATAAAGGAATGATCAATGTGTAGGCTGGAGCTGCTTCG |
| | | mocDreverseKan | GCATTTGTTTAGGGAGGCGAAACGAACTGCGAACCGCCTCCCGGACTACATATGAATATCCTCCTTAGT |
| pMWS112Δ*tmsP* | Δ*tmsP* | tmsPforwardKan | CGATACCGCTCGAACTCCCAGCCTCCTCCCGGGGCACCTGTGTAGGCTGGAGCTGCTTCG |
| | | tmsPreverseKan | GGCGCGGTGTCAGGGGATAGAGCGTTCAGGGCGCGATCGCATATGAATATCCTCCTTAGT |
| pAW19::112Δ*mrtR*(*tetA*) | Δ*mrtR*(*tetA*) | mrtRforwardTet | AGTGACAGTCGCAAGCATACCAATTGGAATGGAGAGCCCATCCCTCTTGACTTATCATTG |
| | | mrtRreverseTet | GTGGAAAATCTGTTCGGACTTAAAGCGGTCTCGAAGAGGGGCGCATGACGTCCGGAGTCT |
| NA[a] | confirmation of the *mocC* mutation | mocCforwardwannercheck | CTCCAGCCGCTCTGAAGAGC |
| | | mocCreversewannercheck | GCCGCATGACAACGCCGAACG |
| NA | confirmation of the *mocD* mutation | mocDforwardwannercheck | GCTCGGTCAAGTCTTCAAGC |
| | | mocDreversewannercheck | CGTGAAGCTGGACTAGGGTG |
| NA | confirmation of the *tmsP* mutation | KT | (Datsenko & Wanner, 2000) |
| | | mocDforwardNdeI | GATCGATCCATATGACAACCCAAAAGACTGATG |
| NA | confirmation of the *mrtR*(*tetA*) mutation | mrtRcheckup | (Wetzel, *et. al*., 2015) |
| | | mrtRcheckdwn | (Wetzel, *et. al*., 2015) |

[a] NA: not applicable

*Supplemental Figure 1. Opines induce transfer of Ti plasmids because they induce transcription of traR.* The operon colored black is an operon that is inducible by the conjugative opine. The *tra* regulon is light grey and the plasmid replication and partitioning genes are dark grey. The genes and proteins involved in regulation of transfer are as follows: *traR*/TraR, black dots on a white background; *traM*/TraM, diagonal stripes; *traI*/TraI, white; and a transcriptional regulator that also senses the conjugative opine, horizontal stripes; Agrobacterium autoinducer (AAI) is indicated by σ, while the conjugative opine is indicated by asterisks (*). Promoters are indicated by "P" and transcriptional direction is indicated by the arrows. (A) Under non-inducing conditions TraM binds to the low levels of TraR that are produced thereby inactivating the transcriptional regulator. (B) Under inducing condition the presence of the conjugative opine induces transcription of an opine-regulated operon, including *traR*. In this manner TraR titrates the effects of TraM and, as long as the population has reached its critical threshold and enough AAI is available, TraR can form active dimers and activate transcription of the *tra* regulon.

*Supplemental Figure 2. TmsPs aligns with the C terminal domain of TraR proteins.* The drawing represents the N terminal AAI binding domain and C terminal DNA binding domain of TraR proteins. The region of TraR that interacts with TraM is also located at the C terminal region. TmsPs and the C terminal region of three TraR proteins were aligned using Clustal W. Invariant residues are purple, conserved resides are blue, while residues that are similar are pink. Above the aligned sequences, the residues of $TraR_{pTiC58}$ important for TraR-TraM interaction (Luo *et al.*, 2000, Qin *et al.*, 2007) are indicated with a black five-

pointed star, while the residues of $TraR_{pNGR234a}$ that are sequence-specific for the TraR-TraM interaction (Chen *et al.*, 2007) are indicated by a white 4-pointed star.

*Supplemental Figure 3. TmsP has three potential start sites*. The nucleotide sequence of *tmsP* and 100 nucleotides upstream of the gene are shown. The three potential start sites are labeled and colored in green, while the stop codon is colored red.

*Supplemental Figure 4. A putative MrtR-binding box is encoded in the intergenic sequence between mrtR and mocC.* (A) *mrtR* and *mocC* are divergently transcribed. The sequence of the nucleotides between the two genes is shown with the start codons for each gene colored in green. The putative MrtR-binding box is a 14 base pair palindromic sequence and is colored red. (B) An alignment of the DNA binding sites for the GntR regulator ExuR from *Escherichia coli* (Rigali *et al.*, 2002), and the putative MrtR box of pAoF64/95.

*Supplemental Figure 5. MocR does not regulate transfer of pAoF64/95.* NTL4 harboring pAoF64/95 and the *mocR* mutant, NTL4(pAoF64/95Δ*mocR*) were assessed for conjugative transfer when grown in AB minimal medium with mannitol (light grey) or mannopine (dark grey) as the primary source of carbon.

## References


Datsenko, K.A. & B.L. Wanner, (2000) One-step inactivation of chromosomal genes in *Escherichia coli* K-12 using PCR products. *Proc. Natl. Acad. Sci. U.S.A.* **97**: 6640-6645.

Chen, G., P.D. Jeffrey, C. Fuqua, Y. Shi & L. Chen, (2007) Structural basis for antiactivation in bacterial quorum sensing. *Proc. Natl. Acad. Sci. U.S.A.* **104**: 16474-16479.

Luo, Z.Q., Y. Qin & S.K. Farrand, (2000) The antiactivator TraM interferes with the autoinducer-dependent binding of TraR to DNA by interacting with the C-terminal region of the quorum-sensing activator. *J. Biol. Chem.* **275**: 7713-7722.

Qin, Y., S. Su & S.K. Farrand, (2007) Molecular Basis of Transcriptional Antiactivation: TraM disrupts the TraR-DNA complex through stepwise interactions. *J. Biol. Chem.* **282**: 19979-19991.

Rigali, S., A. Derouaux, F. Giannotta & J. Dusart, (2002) Subdivision of the helix-turn-helix GntR family of bacterial regulators in the FadR, HutC, MocR, and YtrA subfamilies. *J. Biol. Chem.* **277**: 12507-12515.

Wetzel, M.E., G.J. Olsen, V. Chakravartty & S.K. Farrand, (2015) The *repABC* plasmids with quorum-regulated transfer systems in members of the Rhizobiales divide into two structurally and separately evolving groups. *Genome Biol. Evol.* **7**: 3337-3357.

Supplemental Figure 1

A Uniduced conditions: no conjugative opine

B Induced conditions: conjugative opine

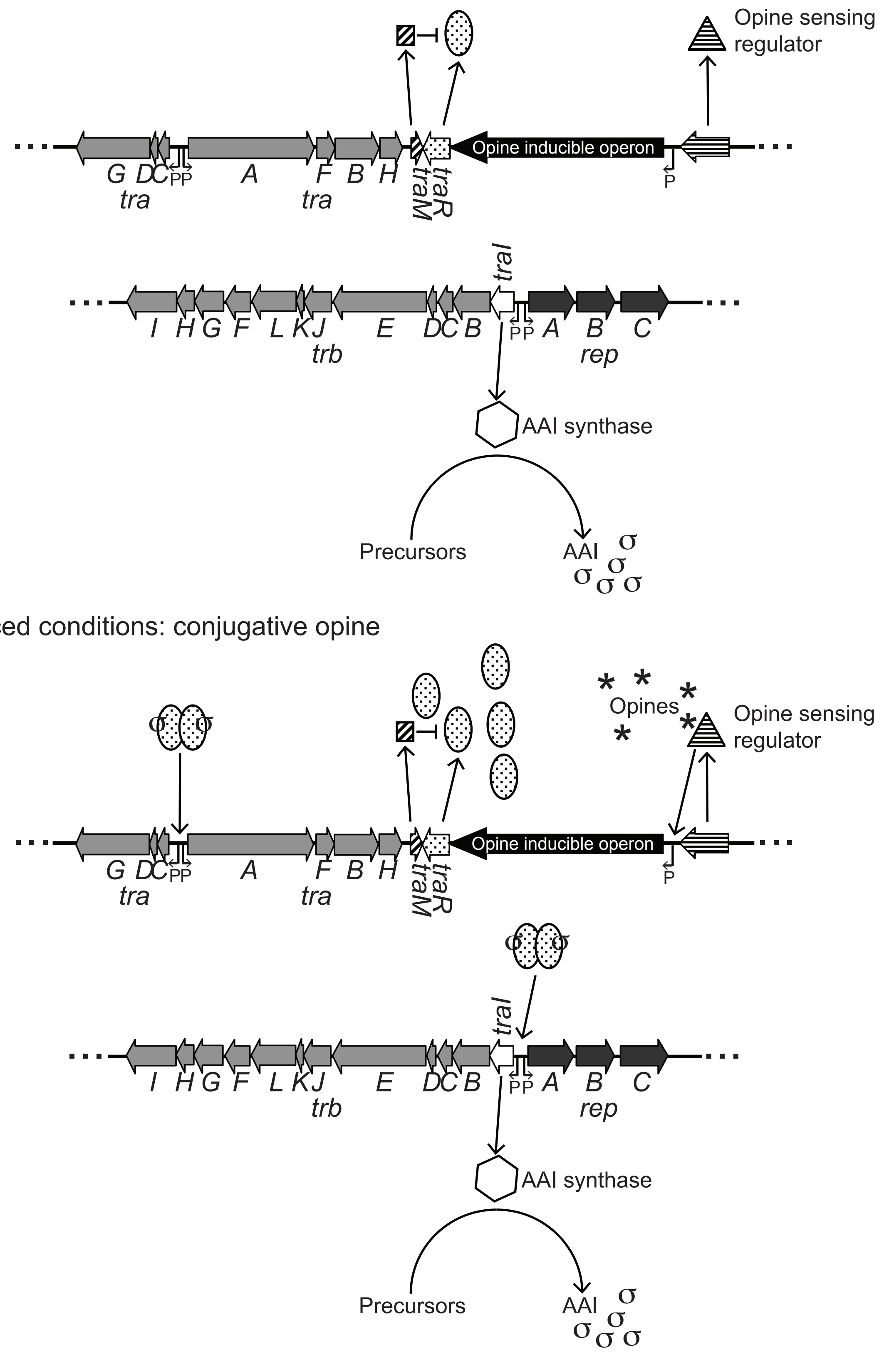

Supplemental Figure 2

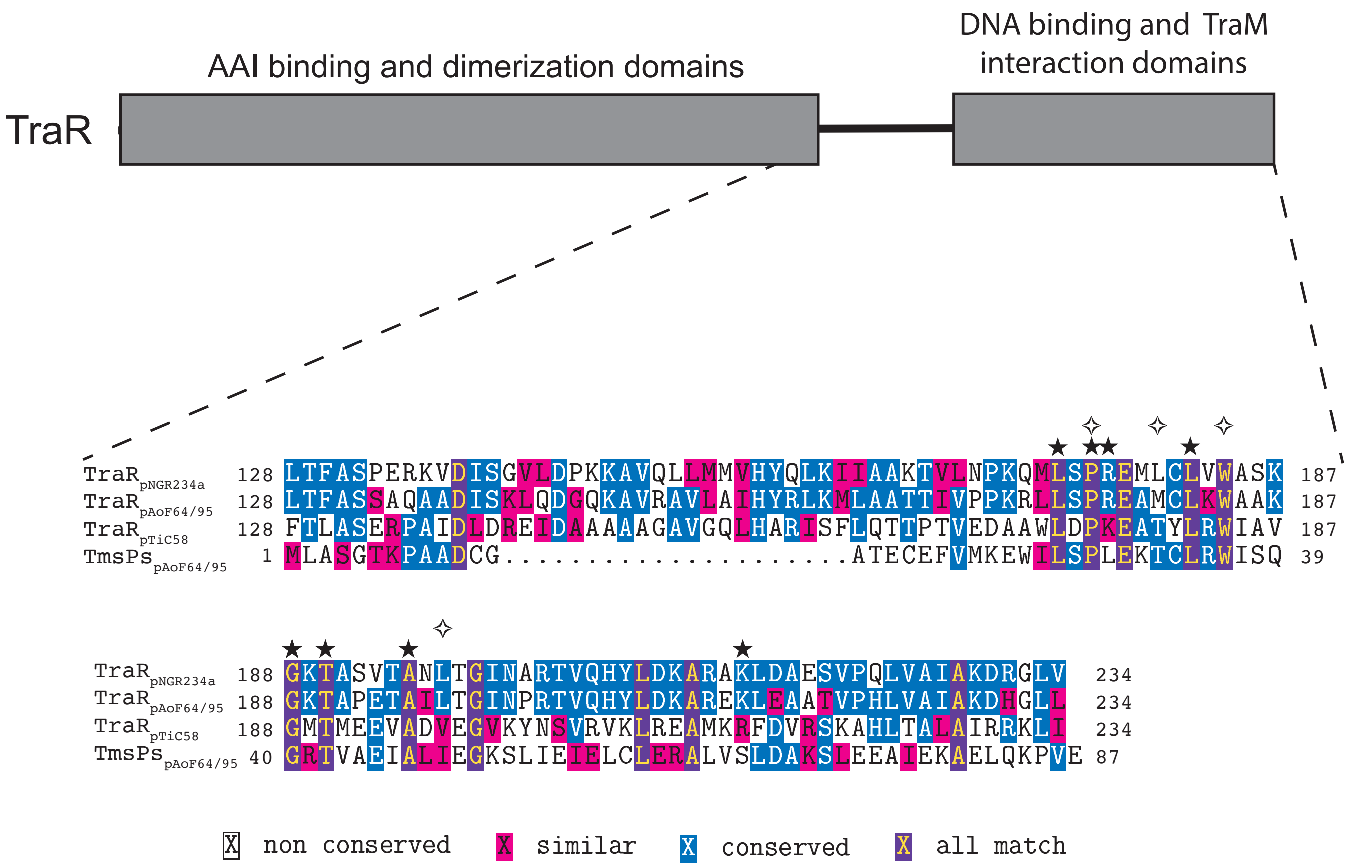

Supplemental Figure 3

```
t gtc cct aac cgt ggc aca cgc tga tcg ctc gga gtg ata ccg att tga t  < 50

gg cat tgc ggg ccg ata ccg ctc gaa ctc cca gcc tcc tcc cgg ggc acc  < 100

 start 1
 |
ttg gct gcc gtc gca cag gtc gcg acg gca gcc act ttt ttc ggg ggc at  < 150
L   A   A   V   A   Q   V   A   T   A   A   T   F   F   G   G   I

          start 2                    start 3
          |                          |
c gcc gcc ttg gct tca acc gac act tca atg ctg gcg tcc ggt acc aaa c  < 200
  A   A   L   A   S   T   D   T   S   M   L   A   S   G   T   K   P

ct gcc gcc gac tgc ggc gca act gag tgc gag ttt gtc atg aag gaa tgg  < 250
   A   A   D   C   G   A   T   E   C   E   F   V   M   K   E   W

ata ctt tcg cca ctt gaa aag acc tgc ctt cgg tgg att tct caa ggc ag  < 300
I   L   S   P   L   E   K   T   C   L   R   W   I   S   Q   G   R

g acc gtc gcc gaa atc gcg ctc att gag ggc aaa agc ctc atc gag atc g  < 350
 T   V   A   E   I   A   L   I   E   G   K   S   L   I   E   I   E

ag ctc tgc ctt gag cgc gcg ctc gta tcg ctt gat gca aaa tcc tta gaa  < 400
   L   C   L   E   R   A   L   V   S   L   D   A   K   S   L   E

                                                 stop
                                                 |
gaa gca att gag aag gcg gag ctt caa aag ccg gtt gaa taa  < 442
E   A   I   E   K   A   E   L   Q   K   P   V   E   *
```

Supplemental Figure 4

A

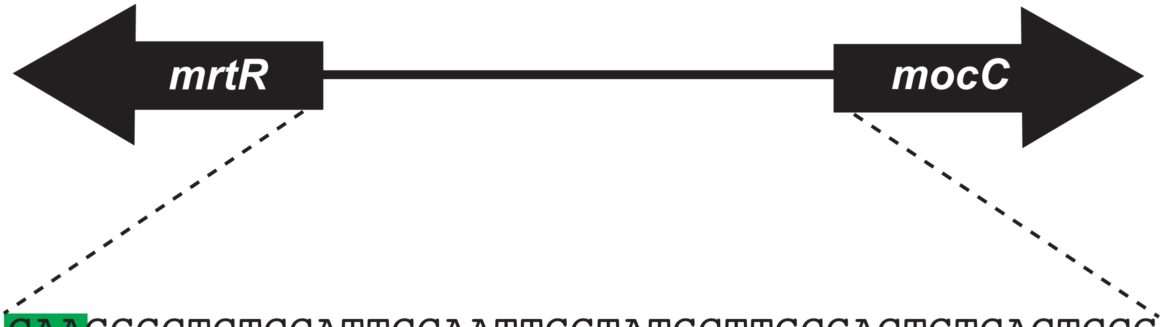


CAAGGGCTCTCCATTCCAATTGGTATGCTTGCGACTGTCACTCCG
CTATTATCGACAAAGAATCCTGCTTTACCACCGCAAAAGAATGCA
AAAGCTTCGAAAAGGCGGGAGCTACGGGGCAATCGCCCGTCGAGG
TCGATAATGCTTGACAGATTGGTATATACCAAGGAAGATTGTAGC
CGCTCTGGTCAAACCGGTGCTGAATGGGCTCCAGCCGCTCTGAAG
AGCGACCATGTTGGAAGTCGCCCAGATATAGCTTTTACTCGGGCG
ACAACTGCCCGCAGACAGCTTCGTTAGAAAGTGAAGAACCATG

Start codons

Putative MrtR-binding box

B

| Regulator | Operator site sequences |
|---|---|
| ExuR | AAATTGGTATA..CCAATTT |
| MrtR | ...TTGGTATATACCAA... |

Supplemental Figure 5

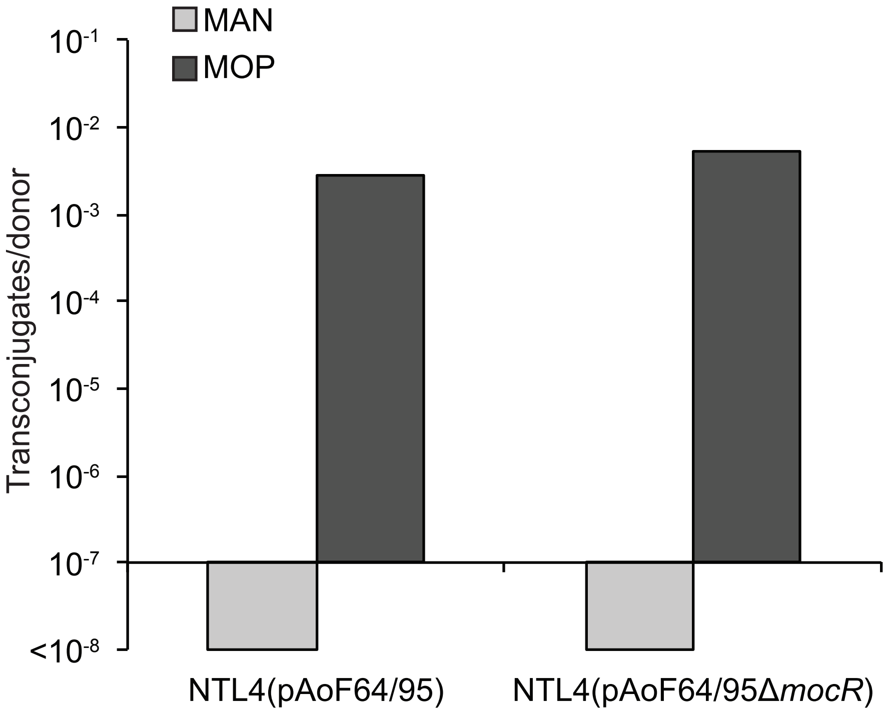

Supplement: Supplementary file 1 [file MBO3-8-e00625-s001.pdf]
